# Comparison of the modulatory effects of three structurally similar potential prebiotic substrates on an in vitro multi-species oral biofilm

**DOI:** 10.1038/s41598-021-94510-z

**Published:** 2021-07-22

**Authors:** Tim Verspecht, Wannes Van Holm, Nico Boon, Kristel Bernaerts, Carlo A. Daep, Naiera Zayed, Marc Quirynen, Wim Teughels

**Affiliations:** 1 Department of Oral Health Sciences, University of Leuven (KU Leuven), Kapucijnenvoer 33, 3000 Leuven, Belgium; 2 Center for Microbial Ecology and Technology (CMET), Ghent University (UGent), Coupure Links 653, 9000 Gent, Belgium; 3 Bio- and Chemical Systems Technology, Reactor Engineering and Safety, Department of Chemical Engineering, University of Leuven (KU Leuven), Leuven Chem&Tech, Celestijnenlaan 200F (bus 2424), 3001 Leuven, Belgium; 4 Colgate-Palmolive Technology Center, 909 River Road, Piscataway, NJ 08854 USA; 5 Faculty of Pharmacy, Menoufia University, Shebeen El-Kom, Egypt; 6 Dentistry, University Hospitals Leuven, Kapucijnenvoer 33, 3000 Leuven, Belgium

**Keywords:** Microbiome, Microbiology, Bacteria, Bacteriology, Biofilms, Microbial communities, Dental diseases, Oral diseases

## Abstract

Previous research identified potential prebiotic substrates for oral health like the structural analogues *N*-acetyl-d-mannosamine (NADM) and *N*-acetyl-d-glucosamine (NADG). The main hypothesis of the current study was twofold. Firstly, it was hypothesized that the modulatory effects of NADM are not limited to changes in multi-species oral biofilm composition, but also include effects on metabolism, virulence, and inflammatory potential. Secondly, the presence and orientation of their *N*-acetyl group could play a role. Therefore, a comparison was made between the effects of NADM, NADG and d-(+)-mannose on multi-species oral biofilms. Besides a beneficial compositional shift, NADM-treated biofilms also showed an altered metabolism, a reduced virulence and a decreased inflammatory potential. At a substrate concentration of 1 M, these effects were pronounced for all biofilm aspects, whereas at ~ 0.05 M (1%_(w/v)_) only the effects on virulence were pronounced. When comparing between substrates, both the presence and orientation of the *N*-acetyl group played a role. However, this was generally only at 1 M and dependent on the biofilm aspect. Overall, NADM was found to have different effects at two concentrations that beneficially modulate in vitro multi-species oral biofilm composition, metabolism, virulence and inflammatory potential. The presence and orientation of the *N*-acetyl group influenced these effects.

## Introduction

Oral diseases such as periodontitis or caries result from complex interplays and feedback loops between pathogenic and commensal microbial species, the host and environmental factors^1–8^. The development of these oral pathologies is characterised by complex, polymicrobial processes originating from a both synergistic and dysbiotic microbiota of which the individual members fulfil well-defined roles that, when combined, give rise to disease^9^. However, in health there is a homeostatic balance between the microbiota, the host and environmental factors^1,3,7^. Preventive and therapeutic strategies in oral healthcare should therefore focus on maintaining and/or restoring the balances between the oral microbiota and the host and within the commensal oral microbiota^1,7,10^. Such approaches could provide valuable alternatives for existing strategies, which often use antimicrobials such as antiseptics or antibiotics. Antimicrobial strategies often result in the a-specific and uncontrolled killing of both commensal and pathogenic bacteria. Furthermore, they could lead to the development of adaptation and resistance to antimicrobial agents, a global healthcare problem that oral healthcare could also be more and more frequently confronted with^11–14^.

In the search for new pro-microbial treatment options, the so-called ‘probiotic approach’, in which live microorganisms are administered in such amounts that they provide a health benefit for the host^15^, gained a lot of interest. Given their broadly shown clinical and microbiological benefits, probiotics for oral health have been used for many years now^16,17^. More recently, the so-called ‘prebiotic approach’, in which substrates that are selectively utilized by host microorganisms are administered and in turn confer a health benefit^18^, was introduced. Even though several in vitro and in vivo studies have explored the use of prebiotics for oral health and reported promising results^19–24^, further in vitro and in vivo research is needed to apply the prebiotic approach in oral healthcare.

In the past few years, potential prebiotic substrates have been identified that modulate in vitro multi-species oral biofilms by stimulation of beneficial/commensal bacteria, resulting in more host-compatible biofilms that carried fewer pathogens, showed reduced virulence gene expression and had less inflammatory potential. *N*-acetyl-d-mannosamine (NADM) was one of the first identified potential prebiotic substrates that could induce a beneficial compositional shift in an in vitro multi-species oral biofilm^22^. Recently, four other potential prebiotic substrates were shown to modulate in vitro multi-species oral biofilms towards a more host-compatible state^24^. More specifically, they induced a shift towards a more health-associated microbiological composition, an altered metabolic profile and an often decreased virulence gene expression and inflammatory potential. One of those substrates, *N*-acetyl-d-glucosamine (NADG), shows high functional and structural similarity with NADM. They both are essential precursors of N-acetyl-neuraminic acid, which constitutes an important component of bacterial cell walls, and have a similar uptake, transport and metabolism^25,26^. Both amino sugars are structural analogues and share the same basic d-(+)-mannose-like sugar structure, but differ in the orientation of their *N*-acetyl group.

The main hypothesis and aim of this study were twofold. Firstly, it was hypothesized that NADM could also have beneficial effects on other aspects of in vitro multi-species oral biofilms. Here, the aim was to investigate whether NADM, apart from its beneficial effect on microbiological composition, also exhibits effects on the metabolic profile, virulence gene expression and inflammatory potential of in vitro multi-species oral biofilms. Secondly, it was hypothesized that the common features of NADM and NADG might play an important role in their observed effects. Here, the aim was to make a comparison between NADM, NADG and d-(+)-mannose to investigate the influence of the presence/absence of the *N*-acetyl group (NADM/NADG vs. d-(+)-mannose) and the orientation of the *N*-acetyl group (NADM vs. NADG) on in vitro multi-species oral biofilm composition, metabolic profile, virulence gene expression and inflammatory potential. All substrates were tested at a higher concentration of 1 M to allow for comparison with the two before-mentioned studies where NADM and NADG were identified as potential prebiotic substrates^22,24^, and at a lower, clinically more relevant concentration of ~ 0.05 M (1%_(w/v)_).

## Results

### Comparison of the effects on multi-species biofilm composition

**Figure 1:**
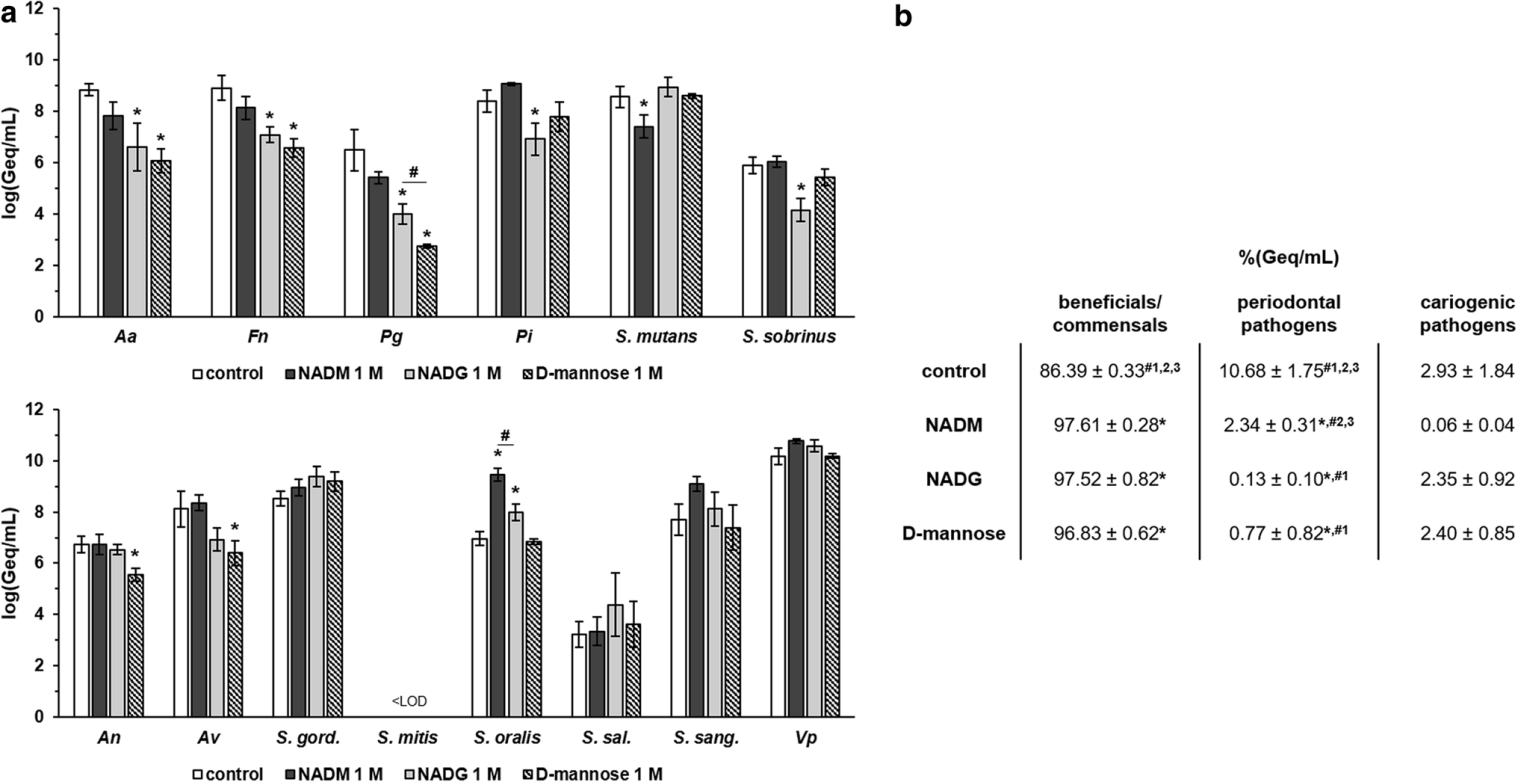
Comparison of the effects of repeated rinsing with NADM, NADG or d-(+)-mannose at 1 M on multi-species biofilm composition. (**Panel a**) Absolute abundances of pathogenic oral species (upper graph) and beneficial/commensal oral species (lower graph) are shown as mean ± SD (n = 3) logarithmic values of the genome equivalents per millilitre (log(Geq/mL)). (**Panel b**) Relative abundances of the different groups (beneficial/commensals, periodontal pathogens, cariogenic pathogens) of bacterial species are shown as mean ± SD (n = 3) percentage of the genome equivalents per millilitre (%(Geq/mL)). All substrates were dissolved in PBS at a concentration of 1 M. Statistically significantly different values when compared to the control (PBS) are marked with ‘*’ (*P* < 0.05 ANOVA + Dunnett’s correction for simultaneous hypothesis testing), statistically significantly different values between two treatment conditions (NADM = condition 1, NADG = condition 2, d-(+)-mannose = condition 3), which were only considered to be relevant if each condition was also significantly different from the control condition, are marked with ‘^**#**^’ (*P* < 0.05, ANOVA + Tukey’s correction for simultaneous hypothesis testing for comparisons between 3 substrates, unpaired two-tailed t-test for comparisons between 2 substrates). *Aa*: *A. actinomycetemcomitans*; *Fn*: *F. nucleatum*; *Pg*: *P. gingivalis*; *Pi*: *P. intermedia*; *An*: *A. naeslundii*; *Av*: *A. viscosus*; *S. gord.*: *S. gordonii*; *S. sal.*: *S. salivarius*; *S. sang.*: *S. sanguinis*; *Vp*: *V. parvula*; LOD: limit of detection (= 2.65 log(Geq/mL)); NADM: *N*-acetyl-d-mannosamine; NADG: *N*-acetyl-d-glucosamine*.*

The effects of NADM, NADG and d-(+)-mannose on multi-species biofilm composition were analysed to assess the influence of the orientation (NADG vs. NADM) and presence/absence (NADG/NADM vs. d-(+)-mannose) of the *N*-acetyl group. Significant differences between two substrate conditions were only considered relevant if they were each also significantly different from the control condition. In terms of absolute numbers (expressed as the log value of the genome equivalents per millilitre; log(Geq/mL)) and at a substrate concentration of 1 M (Fig. 1a, Supplementary Table S1), NADM resulted in a significant decrease (− 1.2 log(Geq/mL)) in *S. mutans* numbers and a significant increase (+ 2.5 log(Geq/mL)) in *S. oralis* numbers compared to the control. NADG resulted in significant decreases in *A. actinomycetemcomitans*, *F. nucleatum*, *P. gingivalis*, *P. intermedia* and *S. sobrinus* numbers (− 1.5 to − 2.5 log(Geq/mL)), and significantly increased *S. oralis* numbers (+ 1.0 log(Geq/mL)). d-(+)-mannose yielded significantly reduced *A. actinomycetemcomitans*, *F. nucleatum*, *P. gingivalis*, *A. naeslundii* and *A. viscosus* numbers (-1.2 to -3.7 log(Geq/mL)). When comparing the numbers of pathogens between the different substrate conditions, only for *P. gingivalis* a significant difference was observed, with d-(+)-mannose resulting in lower amounts compared to NADG (− 1.25 log(Geq/mL))*.* For the beneficials/commensals, a significant difference was only obtained for *S. oralis*, with NADM yielding higher numbers compared to NADG (+ 1.5 log(Geq/mL)).

In terms of relative biofilm composition (expressed as %(Geq/mL)), the control condition resulted in a biofilm harbouring 86.39 ± 0.33% beneficials/commensals, 10.68 ± 1.75% periodontal pathogens and 2.93 ± 1.84% cariogenic pathogens (Fig. 1b). Compared to this, significantly increased abundances of beneficials/commensals were observed following treatment with each of the substrates (97.61 ± 0.28% for NADM, 97.52 ± 0.82% for NADG and 96.83 ± 0.62% for d-(+)-mannose). The periodontal pathogens were significantly decreased for the NADM (2.34 ± 0.31%), NADG (0.13 ± 0.10%) and d-(+)-mannose (0.77 ± 0.82%) conditions, whereas the cariogenic pathogens remained unaffected. For the periodontal pathogens, there was also a significantly decreased abundance compared to NADM for the NADG (0.13 ± 0.10% vs. 2.34 ± 0.31%) and d-(+)-mannose (0.77 ± 0.82% vs. 2.34 ± 0.31%) conditions.

**Figure 2:**
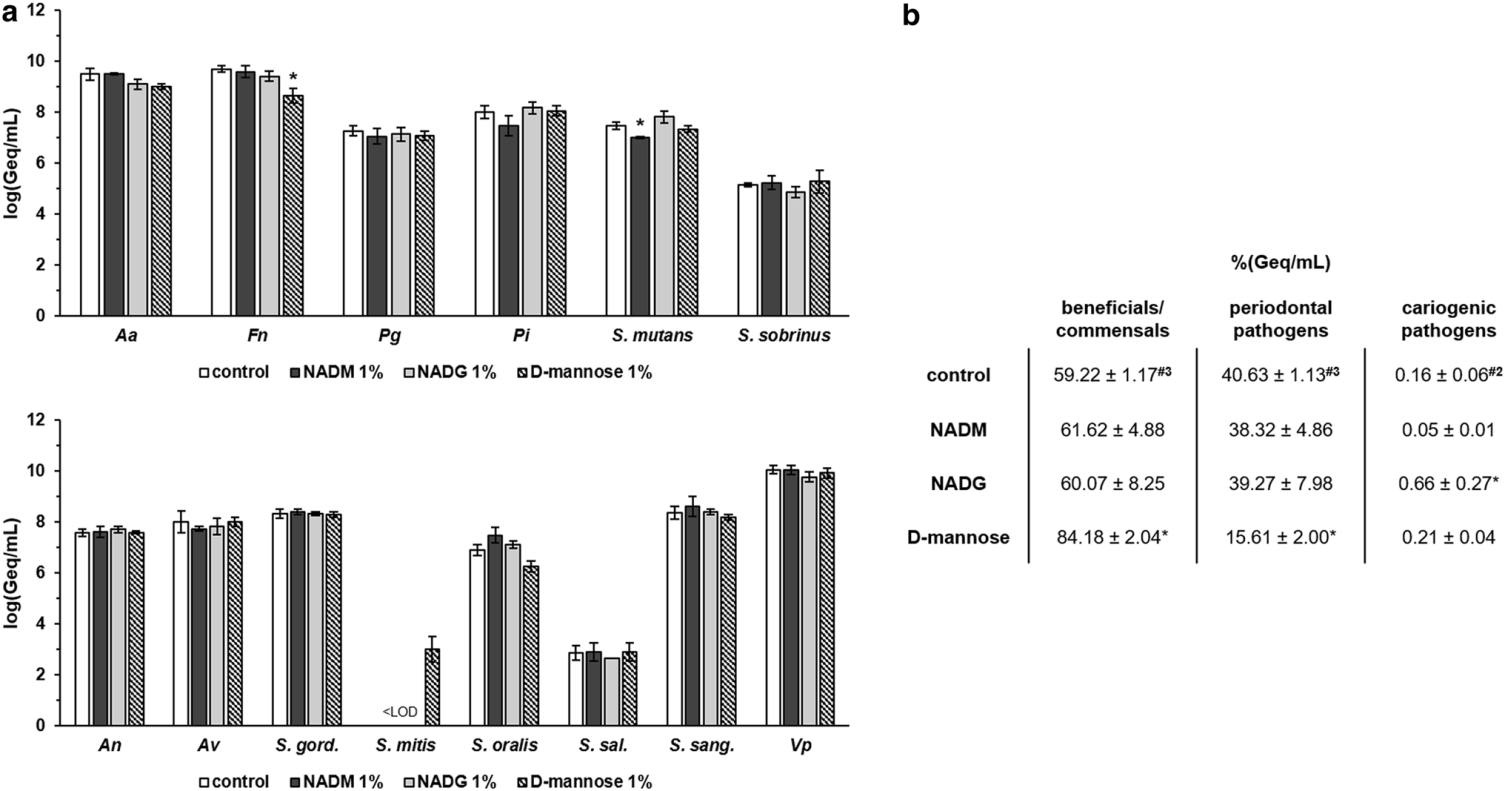
Comparison of the effects of repeated rinsing with NADM, NADG or d-(+)-mannose at ~ 0.05 M (1%_(w/v)_) on multi-species biofilm composition. (**Panel a**) Absolute abundances of pathogenic oral species (upper graph) and beneficial/commensal oral species (lower graph) are shown as mean ± SD (n = 3) logarithmic values of the genome equivalents per millilitre (log(Geq/mL)). (**Panel b**) Relative abundances of the different groups (beneficial/commensals, periodontal pathogens, cariogenic pathogens) of bacterial species are shown as mean ± SD (n = 3) percentage of the genome equivalents per millilitre (%(Geq/mL)). All substrates were dissolved in PBS at a concentration of ~ 0.05 M (1%_(w/v)_) (corresponding molar concentrations: 0.045 M (NADM and NADG) and 0.056 M (d-(+)-mannose)). Statistically significantly different values when compared to the control (PBS) are marked with ‘*’ (*P* < 0.05 ANOVA + Dunnett’s correction for simultaneous hypothesis testing), statistically significantly different values between two treatment conditions (NADM = condition 1, NADG = condition 2, d-(+)-mannose = condition 3), which were only considered to be relevant if each condition was also significantly different from the control conditon, are marked with ‘^**#**^’ (*P* < 0.05, ANOVA + Tukey’s correction for simultaneous hypothesis testing for comparisons between 3 substrates, unpaired two-tailed t-test for comparisons between 2 substrates). *Aa*: *A. actinomycetemcomitans*; *Fn*: *F. nucleatum*; *Pg*: *P. gingivalis*; *Pi*: *P. intermedia*; *An*: *A. naeslundii*; *Av*: *A. viscosus*; *S. gord.*: *S. gordonii*; *S. sal.*: *S. salivarius*; *S. sang.*: *S. sanguinis*; *Vp*: *V. parvula*; LOD: limit of detection (= 2.65 log(Geq/mL)); NADM: *N*-acetyl-d-mannosamine; NADG: *N*-acetyl-d-glucosamine*.*

At a substrate concentration of ~ 0.05 M (1%_(w/v)_) (Fig. 2a, Supplementary Table S2), only *F. nucleatum* and *S. mutans* showed a significant difference with the control, with a − 1.1 log(Geq/mL) and − 0.5 log(Geq/mL) decrease for d-(+)-mannose and NADM, respectively. No relevant significant differences were observed between substrate conditions.

In terms of relative biofilm composition (Fig. 2b), the control condition yielded a biofilm consisting of 59.22 ± 1.17% beneficials/commensals, 40.63 ± 1.13% periodontal pathogens and 0.16 ± 0.06% cariogenic pathogens. Compared to this, d-(+)-mannose resulted in a significantly increased abundance of beneficials/commensals (84.18 ± 2.04%) and a significantly decreased abundance of periodontal pathogens (15.61 ± 2.00%). Furthermore, NADG yielded a significantly increased abundance of cariogenic pathogens (0.66 ± 0.27%). No relevant significant differences between substrate conditions were observed.

### Comparison of the effects on multi-species biofilm organic acid balances

**Figure 3:**
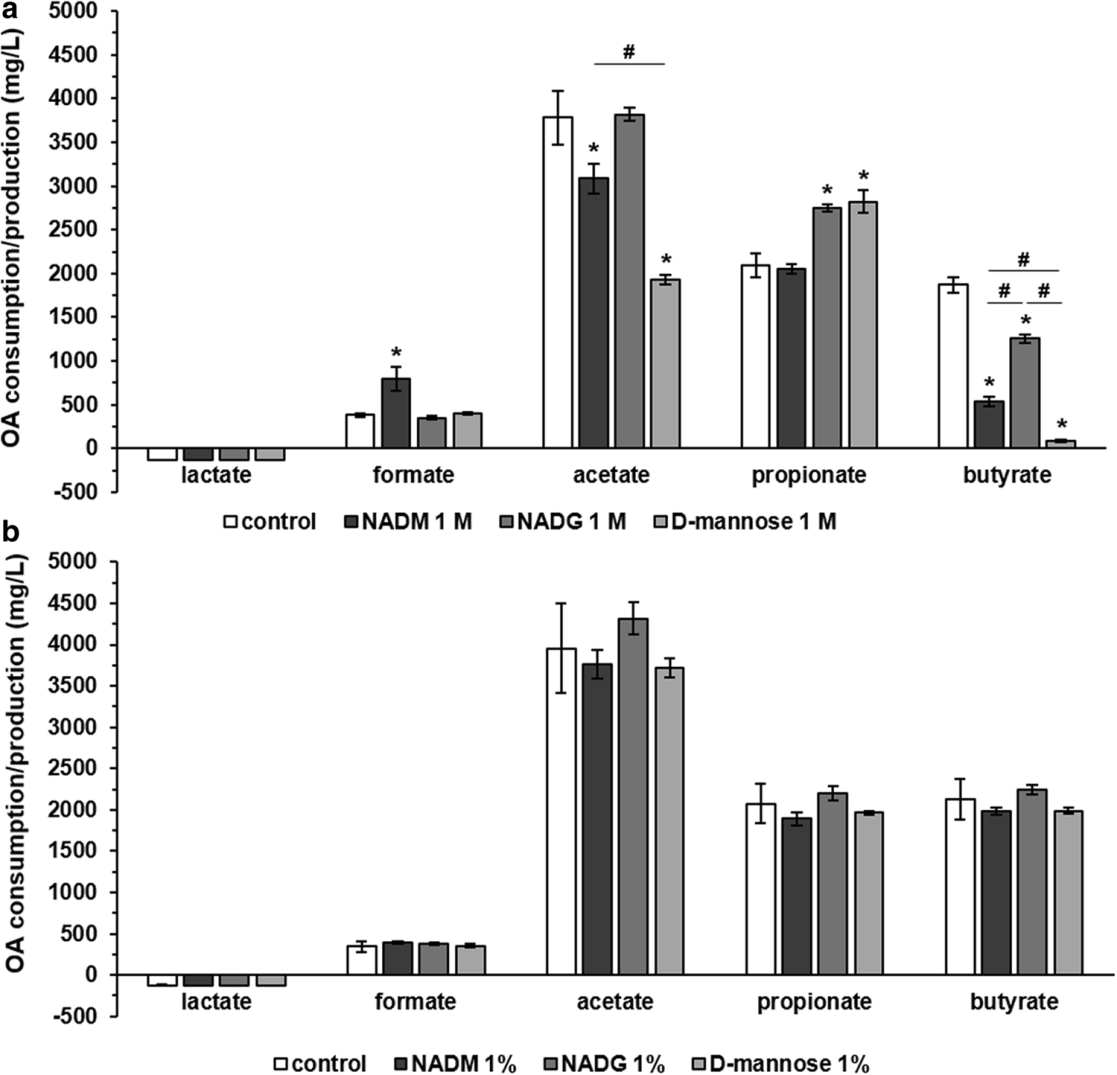
Comparison of the effects of repeated rinsing with NADM, NADG or d-(+)-mannose on multi-species biofilm organic acid production/consumption. Organic acid levels detected in the supernatants of substrate-treated multi-species biofilms are shown as mean ± SD (n = 3) values (mg/L). Values > 0 mg/L represent a net organic acid production, values < 0 mg/L represent a net organic acid consumption. Substrates were dissolved in PBS at a concentration of 1 M (**panel a**) or ~ 0.05 M (1%_(w/v)_) (**panel b**) (corresponding molar concentrations: 0.045 M (NADM and NADG) and 0.056 M (d-(+)-mannose)). Statistically significantly different values when compared to the control (PBS) are marked with ‘*’ (*P* < 0.05, ANOVA + Dunnett’s correction for simultaneous hypothesis testing), statistically significantly different values between two treatment conditions, which were only considered to be relevant if each condition was also significantly different from the control condition, are marked with ‘^**#**^’ (*P* < 0.05, ANOVA + Tukey’s correction for simultaneous hypothesis testing for comparisons between 3 substrates, unpaired two-tailed t-test for comparisons between 2 substrates). OA: organic acid; NADM: *N*-acetyl-d-mannosamine; NADG: *N*-acetyl-d-glucosamine.

To examine the effect of NADM and the influence of the orientation and absence/presence of the *N*-acetyl group on organic acid production and consumption, supernatant samples from substrate-treated or untreated multi-species biofilms were analysed. Significant differences between two substrate conditions were only considered relevant if they were each also significantly different from the control condition. At a substrate concentration of 1 M (Fig. 3, Supplementary Table S3) and when compared to the control, lactate production was unaffected whereas formate production was significantly increased for NADM (791.9 ± 138.27 vs. 384.42 ± 19.24 mg/L). Acetate production was significantly decreased for NADM and d-(+)-mannose (3084 ± 174 and 1934 ± 56 mg/L, respectively, vs. 3779 ± 305 mg/L). Significantly increased propionate production was observed for NADG and d-(+)-mannose (2750 ± 40 and 2821 ± 127 mg/L, respectively, vs. 2094 ± 132 mg/L). Finally, butyrate production was significantly decreased for NADM, NADG and d-(+)-mannose (541 ± 52, 1255 ± 51 and 85 ± 15 mg/L, respectively, vs. 1870 ± 93 mg/L). In terms of relevant significant differences between substrate conditions, an increase in acetate production was observed for NADM compared to d-(+)-mannose (3084 ± 174 vs. 1934 ± 56 mg/L). Butyrate production was significantly lower for NADM and d-(+)-mannose compared to NADG (541 ± 52 and 85 ± 15 vs. 1255 ± 51 mg/L), with in addition d-(+)-mannose yielding lower butyrate production in comparison with NADM (541 ± 52 vs. 85 ± 15 mg/L).

No significant differences could be observed for a substrate concentration of ~ 0.05 M (1%_(w/v)_), both versus the control and when comparing between substrates (Fig. 3, Supplementary Table S3).

### Comparison of the effects on multi-species biofilm virulence gene expression

The expression of 33 virulence genes (for an overview of the associated functions of the corresponding virulence factors: see Supplementary Table S4) from 4 periodontal pathogens was analysed to evaluate the relative virulence of the substrate-treated biofilms. For all three substrates, virulence gene expression was determined relative to the control, and these values were also compared between substrates when this was relevant (Tables 1, 2, 3, 4). Significantly different gene expressions in the substrate-treated biofilms relative to the untreated biofilms were considered to be biologically relevant if their value was more than 2-fold downregulated (2^−ΔΔCt^ < 0.5) or more than 1.5-fold upregulated (2^−ΔΔCt^ > 1.5). Only these results were considered. Furthermore, significant differences between two substrate conditions were only considered relevant if they were each also significantly different from the control condition.

**Table 1:**
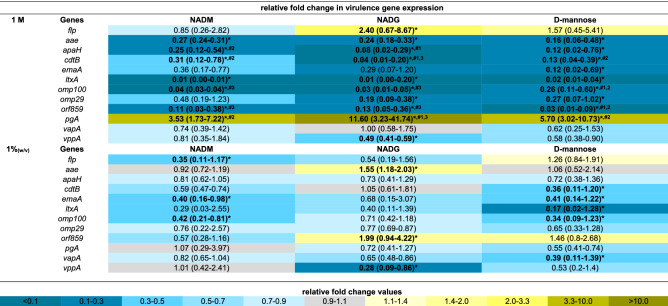
Comparison of the effects of repeated multi-species biofilm rinsing with NADM, NADG or d-(+)-mannose on *A. actinomycetemcomitans* virulence gene expression.

Fold changes in virulence gene expression were determined relative to the control condition through the 2^−ΔΔCt^ method and are shown as the geometric mean (C.I.) (n = 3) of the 2^−ΔΔCt^ values. All substrates were dissolved in PBS at 1 M (upper part) or ~ 0.05 M (1%_(w/v)_) (lower part) (0.045 M (NADM and NADG) and 0.056 M (d-(+)-mannose)). Values between 0 and 1 represent relative downregulation, values > 1 relative upregulation. Statistically significantly values relative to the control (PBS) that are < 0.5 (> twofold downregulated) or > 1.5 (> 1.5-fold upregulated) are considered biologically relevant and shown in bold and marked with ‘*’ (*P* < 0.05, ANOVA + Dunnett’s correction for simultaneous hypothesis testing). For such values, statistically significant differences between two treatment conditions are marked with ‘^**#1**^’ (vs. NADM), ‘^**#2**^’ (vs. NADG), ‘^**#3**^’ (vs. d-(+)-mannose) and shown in bold (*P* < 0.05, ANOVA + Tukey’s correction for simultaneous hypothesis testing for comparisons between 3 substrates, unpaired two-tailed t-test for comparisons between 2 substrates). Statistically significantly different values between treatment conditions were only considered relevant if each condition was also significantly different from the control condition and only such differences are indicated. Color code: magnitude of the fold change in gene expression relative to the control. NADM: *N*-acetyl-*d*-mannosamine; NADG: *N*-acetyl-d-glucosamine; C.I.: 95% confidence interval.

**Table 2:**
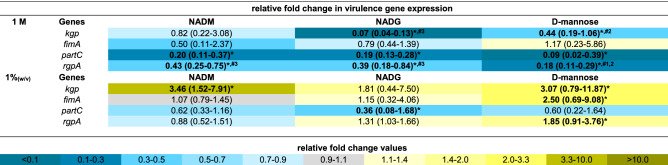
Comparison of the effects of repeated multi-species biofilm rinsing with NADM, NADG or d-(+)-mannose on *P. gingivalis* virulence gene expression.

Fold changes in virulence gene expression were determined relative to the control condition through the 2^−ΔΔCt^ method and are shown as the geometric mean (C.I.) (n = 3) of the 2^−ΔΔCt^ values. All substrates were dissolved in PBS at 1 M (upper part) or ~ 0.05 M (1%_(w/v)_) (lower part) (0.045 M (NADM and NADG) and 0.056 M (d-(+)-mannose)). Values between 0 and 1 represent relative downregulation, values > 1 represent relative upregulation. Statistically significantly different fold changes relative to the control (PBS) that are < 0.5 (> twofold downregulated) or > 1.5 (> 1.5-fold upregulated) are considered biologically relevant and are shown in bold and marked with ‘*’ (*P* < 0.05, ANOVA + Dunnett’s correction for simultaneous hypothesis testing). For such values, statistically significant differences between two treatment conditions are marked with ‘^**#1**^’ (vs. NADM), ‘^**#2**^’ (vs. NADG), ‘^**#3**^’ (vs. d-(+)-mannose) and shown in bold (*P* < 0.05, ANOVA + Tukey’s correction for simultaneous hypothesis testing for comparisons between 3 substrates, unpaired two-tailed t-test for comparisons between 2 substrates). Statistically significantly different values between treatment conditions were only considered relevant if each condition was also significantly different from the control condition and only such differences are indicated. Color code: magnitude of the fold change in gene expression relative to the control. NADM: *N*-acetyl-d-mannosamine; NADG: *N*-acetyl-d-glucosamine; C.I.: 95% confidence interval.

**Table 3:**
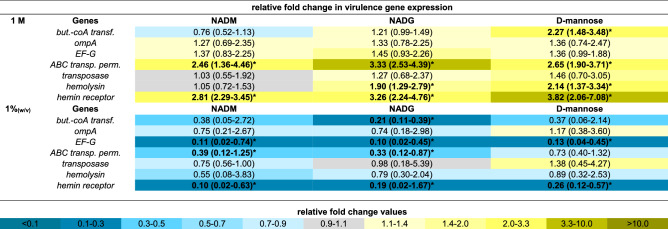
Comparison of the effects of repeated multi-species biofilm rinsing with NADM, NADG or d-(+)-mannose on *F. nucleatum* virulence gene expression.

Fold changes in virulence gene expression were determined relative to the control condition through the 2^−ΔΔCt^ method and are shown as the geometric mean (C.I.) (n = 3) of the 2^−ΔΔCt^ values. All substrates were dissolved in PBS at 1 M (upper part) or ~ 0.05 M (1%_(w/v)_) (lower part) (0.045 M (NADM and NADG) and 0.056 M (d-(+)-mannose)). Values between 0 and 1 represent relative downregulation, values > 1 represent relative upregulation. Statistically significantly different fold changes relative to the control (PBS) that are < 0.5 (> twofold downregulated) or > 1.5 (> 1.5-fold upregulated) are considered biologically relevant and are shown in bold and marked with ‘*’ (*P* < 0.05, ANOVA + Dunnett’s correction for simultaneous hypothesis testing). For such values, statistically significant differences between two treatment conditions are marked with ‘^**#1**^’ (vs. NADM), ‘^**#2**^’ (vs. NADG), ‘^**#3**^’ (vs. d-(+)-mannose) and shown in bold (*P* < 0.05, ANOVA + Tukey’s correction for simultaneous hypothesis testing for comparisons between 3 substrates, unpaired two-tailed t-test for comparisons between 2 substrates). Statistically significantly different values between treatment conditions were only considered relevant if each condition was also significantly different from the control condition and only such differences are indicated. Color code: magnitude of the fold change in gene expression relative to the control. NADM: *N*-acetyl-d-mannosamine; NADG: *N*-acetyl-d-glucosamine; C.I.: 95% confidence interval; but.-coA transf.: butyrate-acetoacetate CoA transferase; ABC transp. perm.: ABC transporter permease.

**Table 4:**
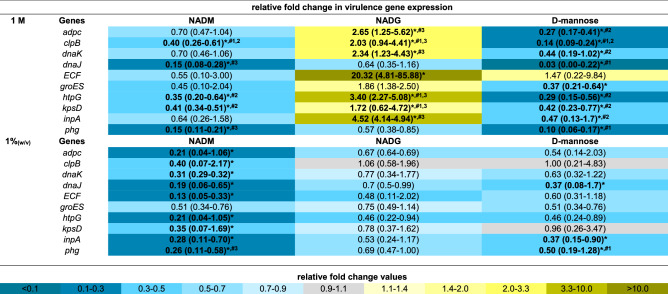
Comparison of the effects of repeated multi-species biofilm rinsing with NADM, NADG or d-(+)-mannose on *P. intermedia* virulence gene expression.

Fold changes in virulence gene expression were determined relative to the control condition through the 2^−ΔΔCt^ method and are shown as the geometric mean (C.I.) (n = 3) of the 2^−ΔΔCt^ values. All substrates were dissolved in PBS at 1 M (upper part) or ~ 0.05 M (1%_(w/v)_) (lower part) (0.045 M (NADM and NADG) and 0.056 M (d-(+)-mannose)). Values between 0 and 1 represent relative downregulation, values > 1 represent relative upregulation. Statistically significantly different fold changes relative to the control (PBS) that are < 0.5 (> twofold downregulated) or > 1.5 (> 1.5-fold upregulated) are considered biologically relevant and are shown in bold and marked with ‘*’ (*P* < 0.05, ANOVA + Dunnett’s correction for simultaneous hypothesis testing). For such values, statistically significant differences between two treatment conditions are marked with ‘^**#1**^’ (vs. NADM), ‘^**#2**^’ (vs. NADG), ‘^**#3**^’ (vs. d-(+)-mannose) and shown in bold (*P* < 0.05, ANOVA + Tukey’s correction for simultaneous hypothesis testing for comparisons between 3 substrates, unpaired two-tailed t-test for comparisons between 2 substrates). Statistically significantly different values between treatment conditions were only considered relevant if each condition was also significantly different from the control condition and only such differences are indicated. Color code: magnitude of the fold change in gene expression relative to the control. NADM: *N*-acetyl-d-mannosamine; NADG: *N*-acetyl-d-glucosamine; C.I.: 95% confidence interval.

At a substrate concentration of 1 M, *A. actinomycetemcomitans* and *P. gingivalis* virulence gene expression was generally significantly downregulated relative to the control for all three substrates (2- to 100-fold for *A. actinomycetemcomitans* and 2.3- to 14.3-fold for *P. gingivalis*) (Tables 1, 2). Noteworthy was the significantly upregulated *A. actinomycetemcomitans* expression of *flp* for the NADG condition (2.4-fold) and *pgA* for all 3 substrate conditions (3.5- to 11.6-fold). In contrast with all this, *F. nucleatum* virulence gene expression was significantly upregulated for all 3 substrates for the ABC transporter permease gene and the hemin receptor gene (2.5- to 3.8 fold), for NADG and d-(+)-mannose for the hemolysin gene (1.9- and 2.1-fold) and for d-(+)-mannose for the butyrate acetoacetate CoA transferase gene (2.3-fold) (Table 3). For *P. intermedia*, a more diverse effect on virulence gene expression was observed, with in general significant decreases for NADM and d-(+)-mannose (2.5- to 33.3-fold) and increases for NADG (2- to 20.3-fold) (Table 4). When only considering relevant significant differences between substrates, NADM usually yielded higher *A. actinomycetemcomitans* gene expressions compared to NADG (Table 1). The opposite was observed for *flp* and *pgA*. d-(+)-mannose resulted in higher *omp100* expression compared to NADM and NADG, whereas the opposite was observed for *orf859*. *cdtB* expression was higher for d-(+)-mannose compared to NADG, whereas for *pgA* this was the opposite. For *P. gingivalis*, differences were limited, with NADG resulting in lower *kgp* expression compared to d-(+)-mannose, and d-(+)-mannose resulting in lower *rgpA* expression compared to NADM and NADG (Table 2). For *F. nucleatum*, no relevant significant differences between substrate conditions were observed (Table 3). For *P. intermedia*, the expression of most genes was significantly higher for NADG when compared to NADM and d-(+)-mannose. *clpB*, *dnaJ* and *phg* expression was lower for d-(+)-mannose in comparison with NADM (Table 4).

At a substrate concentration of ~ 0.05 M (1%_(w/v)_), *A. actinomycetemcomitans* gene expression was significantly decreased relative to the control for NADM in the case of *flp*, *emaA* and *omp100* (2.4- to 2.9-fold), for d-(+)-mannose in the case of *cdtB*, *emaA*, *ltxA*, *omp100* and *vapA* (2.4- to 5.9-fold), whereas for NADG it was increased in the case of *aae* and *orf859* (1.5- and twofold) and decreased in the case of *vppA* (3.6-fold) (Table 1). For *P. gingivalis* and *F. nucleatum*, the observed pattern of significant differences in gene expression relative to the control was generally opposed to the one observed for a substrate concentration of 1 M (Tables 2, 1). When significantly affected, *F. nucleatum* gene expression was decreased (2.6- to 10-fold) and *P. gingivalis* gene expression increased (1.8- to 3.5-fold), except for *partC* expression, which was decreased (2.8-fold) for NADG. Relative *P. intermedia* virulence gene expression was generally downregulated for NADM (2.5- to 7.7-fold), unaffected for NADG and downregulated for d-(+)-mannose for 3 genes (2- to 2.7-fold) (Table 4). When considering only relevant significant differences between substrates, no differences were observed for *A. actinomycetemcomitans*, *P. gingivalis* and *F. nucleatum* (Tables 1, 2, 3). For *P. intermedia*, *phg* expression was lower for NADM compared to d-(+)-mannose (Table 4).

### Comparison of the effects on multi-species biofilm inflammatory potential

The expression of five inflammatory mediator genes in human oral keratinocytes (HOKs) exposed to substrate-treated or untreated biofilms was analysed to evaluate the relative inflammatory potential of substrate-treated biofilms. In addition, these values were also compared between substrates when this was relevant. Significantly different gene expressions relative to HOKs exposed to untreated biofilms were considered biologically relevant if their value was more than 2-fold downregulated (2^−ΔΔCt^ < 0.5) or more than 1.5-fold upregulated (2^−ΔΔCt^ > 1.5) and only these results were considered. Furthermore, significant differences between two substrate conditions were only considered relevant if they were each also significantly different from the control condition. Finally, absolute IL-8 levels in the cellular supernatant were determined as well.

**Figure 4:**
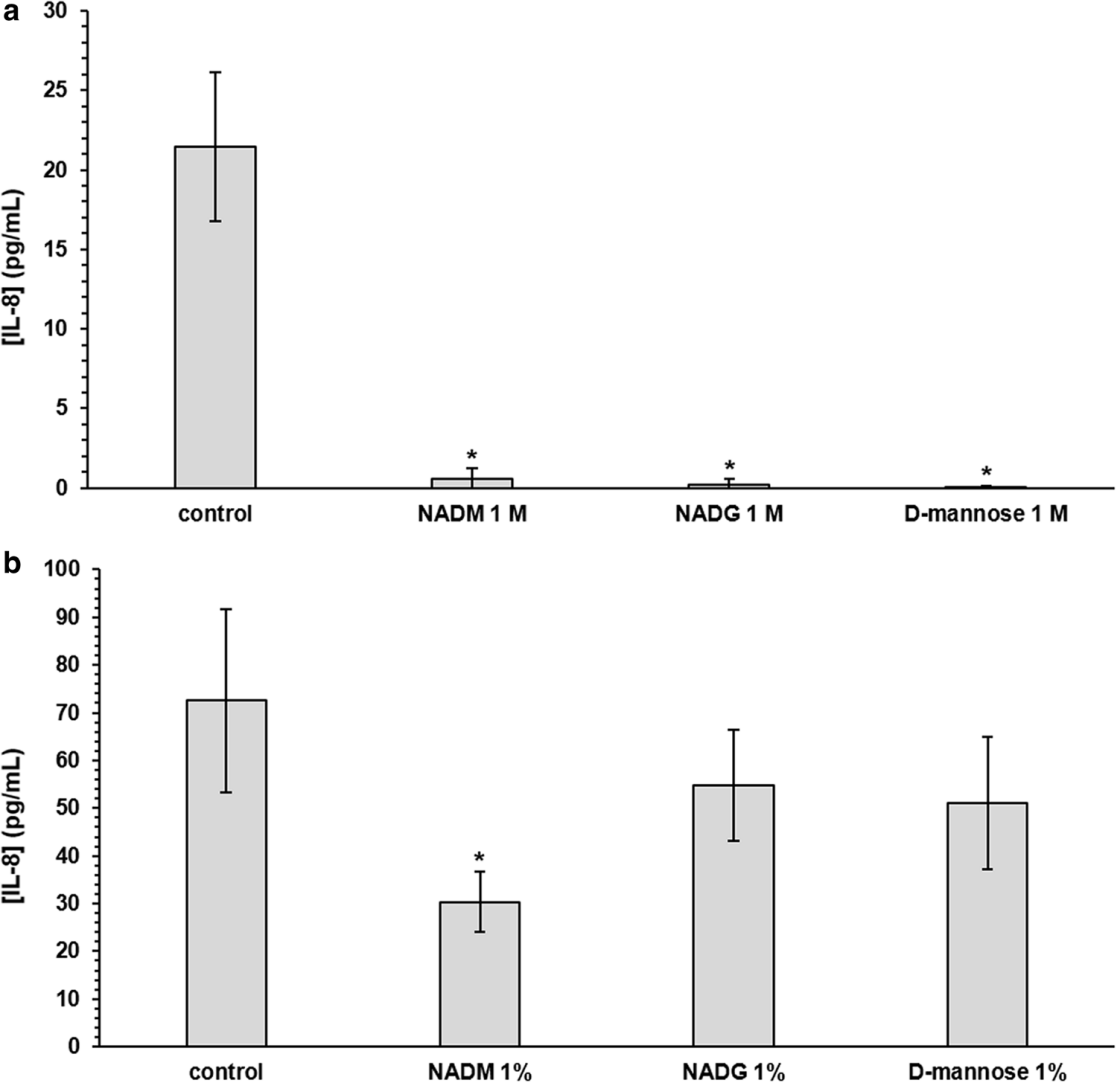
Comparison of the effects of repeated rinsing with NADM, NADG or d-(+)-mannose on multi-species biofilm inflammatory potential. IL-8 levels detected in the supernatants of human oral keratinocytes (HOK-18A) cultures exposed to substrate-treated multi-species biofilms are shown as mean ± SD (n = 3) values (pg/mL). Substrates were dissolved in PBS at a concentration of 1 M (**panel a**) or ~ 0.05 M (1%_(w/v)_) (**panel b**) (corresponding molar concentrations: 0.045 M (NADM and NADG) and 0.056 M (d-(+)-mannose)). Statistically significantly different values when compared to the control (PBS) are marked with ‘*’ (*P* < 0.05, ANOVA + Dunnett’s correction for simultaneous hypothesis testing), statistically significantly different values between two treatment conditions, which were only considered to be relevant if each condition was also significantly different from the control condition, are marked with ‘^**#**^’ (*P* < 0.05, ANOVA + Tukey’s correction for simultaneous hypothesis testing for comparisons between 3 substrates, unpaired two-tailed t-test for comparisons between 2 substrates). NADM: *N*-acetyl-d-mannosamine; NADG: *N*-acetyl-d-glucosamine.

Exposure of HOKs to substrate-treated biofilms (substrate concentration of 1 M) resulted in a decreased IL-8 gene expression (7.1-fold to 10-fold) relative to HOKs exposed to untreated biofilms (Supplementary Table S5). The relative expression of the other genes was generally unaffected, except for the MMP-8 gene, for which it was increased for the NADM condition (1.7-fold). When comparing between substrates, no relevant significant differences were observed. Absolute IL-8 levels were significantly decreased (36-fold to 357-fold) for all three substrate conditions compared to the control (Fig. 4, Supplementary Table S6). No relevant significant differences in IL-8 levels were observed when comparing between substrates.

Exposure of HOKs to substrate-treated biofilms (substrate concentration of ~ 0.05 M (1%_(w/v)_)) did generally not yield significant differences in gene expression relative to HOKs exposed to untreated biofilms, except for a decreased MMP-8 gene expression for the d-(+)-mannose condition (2.5-fold) (Supplementary Table S5). No relevant significant differences were observed when comparing between substrates. This was also the case for IL-8 levels when compared to the control condition or between substrates, except for the NADM condition, which showed reduced IL-8 levels compared to the control (2.4-fold) (Fig. 4, Supplementary Table S6).

## Discussion

Over the past years, research groups have started to focus on the modulation of the commensal oral microbiota as a potential strategy for the prevention and treatment of oral diseases^19–22,24,27^. In this study, two important hypotheses that emerged from previous research were investigated. Firstly, this study shows that a previously identified potential prebiotic substrate, NADM, does not only modulate in vitro multi-species oral biofilms so that they become more host-compatible in terms of microbiological composition, as previously shown^22^, but also modulates other important biofilm aspects. Biofilms repeatedly rinsed with NADM showed an altered metabolic activity, a downregulated expression of a selection of virulence genes and a decreased inflammatory potential towards human oral keratinocytes. At a concentration of 1 M, NADM had pronounced effects on all four biofilm aspects, whereas at a concentration of ~ 0.05 M (1%_(w/v)_), NADM mainly had pronounced effects on virulence gene expression. This study thus further strengthened the candidacy of NADM as a potential prebiotic for oral health. Secondly, a comparison was made between the two previously identified potential prebiotic substrates NADM and NADG^22,24^, and d-(+)-mannose, to investigate the effects of the presence/absence and the orientation of the *N*-acetyl group on the four above-mentioned biofilm aspects. Both the presence/absence and the orientation were sometimes found to play a role when considering compositional, metabolic and virulence changes. However, this was generally only at a substrate concentration of 1 M and often dependent on the biofilm aspect under consideration. Since d-(+)-mannose also had beneficial effects, it can be stated that the *N*-acetyl group is not essential or required to achieve certain biofilm modulatory effects. However, to achieve certain other effects, both the presence and orientation of the *N*-acetyl group played a role. Altogether, the novelty of this study is twofold. Firstly, it broadens the range of modulatory effects the previously identified potential prebiotic substrate NADM has on in vitro 14-species oral biofilms. Secondly, it identifies differences between the modulatory effects of NADM and NADG, two previously identified potential prebiotic substrates, on in vitro multi-species oral biofilms.

Evaluation of the substrates at two distinct concentrations, a low one (~ 0.05 M (1%_(w/v)_)) and a high one (1 M), was an important experimental factor that allowed for comparison with the two studies preceding this work^22,24^. Like it is the case for many therapeutic or prophylactic formulations, prebiotics are characterized by a dose-dependency of their effects, which only become noticeable above a certain threshold concentration^22,28–30^. In the previous in vitro study by Slomka and co-workers where NADM was shown to have potential prebiotic effects on multi-species oral biofilm composition, the effects were most pronounced for concentrations near the 1 M concentration used in the current study. In the study by Verspecht et al. where NADG was identified as a potential prebiotic substrate, the effects were also most pronounced for a concentration of 1 M. Such a high concentration can be useful to provide a certain in vitro proof-of-concept, but commercial oral healthcare products would eventually require much lower concentrations of the compounds in their formulation. Therefore, the more realistic concentration of ~ 0.05 M (1%_(w/v)_) was included as well.

One of the ultimate goals of microbiota modulation-based interventions is to restore or maintain the homeostatic balance between beneficial/commensal and potentially pathogenic microbial species. In this study, repeated rinsing of the biofilms with NADM at 1 M caused a clear shift in biofilm composition towards a more health-associated one^31–33^, with decreased proportions of periodontal and cariogenic pathogens and increased proportions of beneficial/commensal species. This corresponds with what was previously reported for NADM by Slomka and co-workers, who showed that repeated exposure of an in vitro multi-species oral biofilm to NADM resulted in a biofilm composition of > 96% beneficial/commensal species^22^. However, such pronounced compositional changes were lacking at a substrate concentration of ~ 0.05 M (1%_(w/v)_). Most likely, this is due to the concentration dependency of (potential) prebiotic substrates, something which has also been reported before^22,29,34^. In addition, this could also be because of the relatively short treatment period used in this study. Further investigation is required to obtain conclusive results regarding this specific matter.

Because of the well-known association between metabolic activity and microbiological composition and interactions^35–38^, this study also evaluated the effects of NADM on the organic acid balances in the biofilm supernatant. Given the diversity in species of the biofilm model used in this study, a complex interplay between the different metabolic networks of the species is to be expected. The most common metabolic pathways in oral bacteria and the resulting metabolites have been previously described by Takahashi and coworkers^35,38^. Being an amino sugar, NADM is primarily metabolized through saccharolytic pathways, providing a nitrogen and carbon source^39–41^. Rinsing the biofilms with NADM at 1 M induced changes in organic acid balances that could be explained by some of the compositional changes. A possible explanation for the lactate depletion, one of the main metabolites of a saccharolytic streptococcal metabolism, and increased formate levels might be the tendency of increased *Veillonella parvula* numbers. *Veillonella* species are usually highly abundant in oral biofilms and utilize lactate as an energy source by for instance metabolizing it into formate, acetate and propionate^31,33,38^. Another remarkable observation was the decrease in butyrate levels, which can be attributed to the generally decreased abundances of the periodontal pathogens. Especially this last metabolic change can be considered as a favourable health-associated change since butyrate produced by periodontal pathogens is associated with inflammatory processes that take place during periodontal diseases^42,43^. Similar to what was observed at the compositional level, no detectable changes in organic acid balances occurred when NADM was tested at ~ 0.05 M (1%_(w/v)_), which is perhaps not surprising given the strong association between compositional and metabolic changes.

Inflammation caused by a virulent, dysbiotic microbiota is one of the main hallmarks of oral diseases^44–48^. Therefore, the expression of a selection of well-known virulence genes from the periodontal pathogens incorporated in the biofilm model was evaluated. In most cases, virulence gene expression was often strongly (2- up to 100-fold) downregulated in biofilms repeatedly rinsed with NADM, and this for both concentrations tested. These genes encode a wide range of virulence factors involved in a variety of disease-associated processes such as immune evasion, host tissue degradation, cytotoxicity and cell adhesion and invasion (Supplementary Table S4)^49–54^. Numerous in vitro and in vivo studies investigating dysbiotic oral biofilms have reported upregulated expression of these or similar virulence genes^44,46,55,56^. Consequently, one can conclude that NADM theoretically also exhibits a beneficial, health-associated modulatory effect on the virulence properties of multi-species oral biofilms. Worth highlighting is the fact that an altered virulence gene expression was also observed for the ~ 0.05 M (1%_(w/v)_) condition, despite the lack of compositional or metabolic changes for this substrate concentration. This has also been described for subinhibitory concentrations of certain antimicrobials, which were unable to affect bacterial growth but still caused pronounced changes in virulence gene expression^57–59^. Overall, NADM treatment showed to have beneficial effects on virulence gene expression, which were highly dependent on the substrate concentration, the bacterial species and the genes under consideration.

As mentioned before, inflammation plays a key role in the initiation and progression of periodontal diseases^48^. Biofilms treated with NADM at 1 M showed a pronounced reduction of their inflammatory potential towards biofilm-exposed human oral keratinocytes in terms of IL-8 gene expression levels and absolute IL-8 levels. In addition, keratinocytes exposed to biofilms treated with NADM at ~ 0.05 M (1%_(w/v)_) also showed a decrease in absolute IL-8 levels. These keratinocytes are known to produce high levels of IL-8 during the onset and progression of periodontal disease, with IL-8 subsequently acting as a strong attractant and activator of neutrophils^55,60,61^. The reduced IL-8 levels can thus be considered as favourable, as this could breach the self-sustaining feedback loop between dysbiosis and inflammation by which periodontal disease is characterized. Finally, there could also still be a more indirect effect on the inflammatory response, for instance through the before-mentioned changes in virulence gene expression.

Altogether, the data support the hypothesis that the biofilm modulatory effects of NADM are not limited to compositional changes, but also include changes in microbial function (i.e. metabolic activity and virulence) and in interaction with the host (i.e. inflammatory potential). A similar general conclusion was previously drawn for NADG^24^. NADM and NADG both are essential precursors of N-acetylneuraminic acid, an important component in bacterial cell walls (peptidoglycan) and bacterial capsules (polysialic acid), and share their main uptake and transport mechanisms and metabolization processes^25,26,62,63^. Both amino sugars share the same basic d-(+)-mannose-like sugar structure, but differ in the orientation of their *N*-acetyl group. This study attempted to investigate whether the presence/absence and the orientation of the *N*-acetyl group plays a role in the observed biofilm modulatory effects. In some cases, differences between NADM, NADG and d-(+)-mannose were observed, indicating that both the presence and the orientation might have an influence. The data show that all three substrates have certain beneficial modulatory effects on in vitro multi-species oral biofilms. Sometimes these effects are only observed for one or two substrate(s), or sometimes certain effects are less or more pronounced. When there seemed to be an influence of the absence/presence or orientation of the *N*-acetyl group, this was highly dependent on the specific biofilm aspect under investigation. This is well-illustrated by the differences in effects on virulence gene expression, which were highly dependent on the bacterial species, virulence gene and substrate concentration. There are numerous examples in nature of bioactive molecules that have similar molecular structures but differ in their biological effects, ranging from subtle, unharmful differences to completely opposed effects^64–66^. The overall beneficial effects achieved for all three substrates raise the question whether combining two or three substrates could result in synergistic effects. Although this was not investigated in the current study, it is unlikely this would be the case. A study on the transport of NADM and NADG in *Escherichia coli* reported strong inhibition of NADM and NADG uptake by mannose and both amino sugars also strongly affected each other’s uptake^26^.

The differences and limitations of this type of in vitro research in comparison with the real-life situation have already been broadly discussed and addressed in the previous study by Verspecht et al.^24^. The experimental set-up of this study was designed to deal with several of these limitations and differences. For instance, biofilms were grown on hydroxyapatite disks to simulate the tooth surface, whereas the rinsing protocol simulated repeated exposure to the substrates as would be the case when using mouth rinses or brushing the teeth. Future research should address other aspects that were left untouched in the current study, like the inclusion of other inflammatory models or looking into actual levels of virulence factors. In vivo studies will eventually be required to determine the clinical potential of these potential prebiotic substrates.

In conclusion, this study showed that the previously identified potential prebiotic substrate NADM has distinct effects at two different concentrations that modulate in vitro multi-species oral biofilms in such a way that they become more host-compatible. These effects are not only limited to compositional changes but also include effects on metabolism, virulence and inflammatory potential. Repeatedly rinsing established biofilms with NADM at the highest concentration tested (1 M) caused pronounced compositional, metabolic, virulence and inflammatory changes, whereas at the lowest concentration tested (~ 0.05 M (1%_(w/v)_)) mainly changes in virulence gene expression were observed. Furthermore, this study investigated whether the presence/absence and orientation of the *N*-acetyl group present in both previously identified potential prebiotic substrates NADM and NADG influence the biofilm modulatory effects. Sometimes both the presence and orientation influenced these effects, but this was highly dependent on the biofilm aspect under investigation. Altogether, this study further strengthens the candidacy of NADM as a potential prebiotic for oral health. However, when comparing between the effects of the three substrates, the conclusions were less straightforward. Since d-(+)-mannose also had beneficial effects, it can be stated that the *N*-acetyl group is not essential or required to achieve certain biofilm modulatory effects. However, to achieve certain other effects, both the presence and orientation of the *N*-acetyl group played a role.

## Materials and methods

### Bacterial strains, media and culture conditions

Following bacterial strains were used as representative oral pathogens: *Aggregatibacter actinomycetemcomitans* ATCC 43718, *Fusobacterium nucleatum* ATCC 10953, *Porphyromonas gingivalis* ATCC 33277, *Prevotella intermedia* ATCC 25611, *Streptococcus mutans* ATCC 25175 and *Streptococcus sobrinus* ATCC 33478. *Actinomyces naeslundii* ATCC 51655, *Actinomyces viscosus* ATCC 15987, *Streptococcus gordonii* ATCC 49818, *Streptococcus mitis* DSM 12643, *Streptococcus oralis* DSM 20627, *Streptococcus salivarius* TOVE-R, *Streptococcus sanguinis* LMG 14657 and *Veillonella parvula* DSM 2008 were used as representative commensal/beneficial oral bacterial strains. Bacteria were maintained on blood agar plates consisting of Blood agar Base I (Oxoid Ltd, Basingstoke, UK) supplemented with 1 mg/mL menadione (Calbiochem-Novabiochem, La Jolla, USA), 5 mg/mL hemin (Sigma-Aldrich Co, St. Louis, USA) and 5% sterile horse blood (E&O Laboratories Ltd, Bonnybridge, Scotland). Detailed culture and incubation conditions were described previously by Slomka and co-workers^22^.

### Bioreactor-derived multispecies community

A 14-species community was established in a Biostat B Twin 1L bioreactor (Sartorius Stedim Biotech GmbH, Goettingen, Germany) with controlled environmental conditions as previously described by Slomka and co-workers^22^.

### Substrates

*N*-acetyl-d-glucosamine, *N*-acetyl-d-mannosamine and d-(+)-mannose (all Sigma-Aldrich Co, St. Louis, USA) were dissolved in phosphate-buffered saline (PBS) with pH adjusted to 5.7 using citric acid, followed by filter sterilization. Substrates were tested at concentrations of 1 M and ~ 0.05 M (1%_(w/v)_), the latter respectively corresponding to 0.045 M (NADG and NADM) and 0.056 M (d-(+)-mannose).

### Multi-species biofilm rinsing assays

For the biofilm rinsing assays, a bioreactor sample was diluted (1:10) in fresh modified Brain Heart Infusion (BHI) medium^67^ and added to a 24-well plate (2 mL/well). The Amsterdam Active Adhesion model^68^ was used to grow biofilms vertically on Calcium Deficient Hydroxyapatite (CAD-HA) disks (Hitemco Medical, Old Bethpage, USA). Biofilms were grown under micro-aerophilic conditions (6% O_2_, 7% CO_2_, 7% H_2_, 80% N_2_) during 24 h (37 °C, 170 rpm). Next, disks containing the established biofilms were rinsed 3 times a day for 3 min (RT, 250 rpm), during 3 consecutive days, by transferring the lid containing the disks to a new 24-well plate containing the appropriate substrate solutions (2 mL/well). PBS (pH 5.7) without substrate supplementation was used as a negative control. After each rinsing step, disks were shortly dip-rinsed in a new 24-well plate containing fresh modified BHI medium (2 mL/well) to remove remaining substrate traces. Subsequently, the lid was transferred to another 24-well plate containing fresh modified BHI medium (2 mL/well), followed by incubation (micro-aerophilic, 37 °C, 170 rpm) until the next rinsing step. The morning after the final rinsing step on the third day, disks containing treated and untreated biofilms were dip-rinsed in PBS (pH 7.4) to remove unattached cells, followed by bacterial DNA or RNA extraction or by biofilm challenge of human oral keratinocytes (see lower). All experiments were repeated on three different days.

### Bacterial DNA extraction and quantification

Biofilm-coated disks were dip-rinsed in PBS (pH 7.4) to remove unattached cells, after which biofilms were disrupted. Next, bacterial cells were harvested and DNA was extracted only from living bacteria using propidium monoazide (PMA), as described previously^22^. Each bacterial species was quantified by means of a quantitative PCR (qPCR) assay as described earlier^22^, with species-specific primers and probes identical to those listed by Herrero et al^69^.

### Organic acid analysis

The supernatant of the multi-species biofilm cultures was collected and filter sterilized, after which concentrations of organic acids were measured using a 761 Compact Ion Chromatograph (Metrhohm, Switzerland) with a Metrosep Organic acids 250/7.8 column and a Metrosep Organic acids Guard/4.6 guard column. The eluent consisted of 1 mM H_2_SO_4_ at a flow rate of 0.8 mL min^−1^. The production/consumption of organic acids was calculated as the organic acid concentrations detected in the filter-sterilized supernatant, minus the organic acid concentrations detected in sterile modified BHI medium.

### Bacterial RNA extraction and virulence gene expression analysis

Biofilm-coated disks were dip-rinsed in PBS (pH 7.4) to remove unattached cells, after which bacterial RNA was extracted according to a mechanical disruption and acid phenol–chloroform extraction as described by Vandecasteele et al.^70^_**,**_ combined with the RNeasy Mini Kit (Qiagen, Hilden, Germany) according to the manufacturer’s protocol. After assessment of RNA quality and integrity, a concentration-dependent normalization of all RNA samples was performed. Next, RNA was converted to complementary DNA (cDNA) using the PrimeScript 1^st^ strand cDNA Synthesis Kit (Takara, Shiga, Japan) according to the manufacturer’s protocol. Expression of bacterial virulence genes was analysed by SYBR RT-qPCR and normalized for bacterial housekeeping gene expression (species-specific 16S rRNA or other genes). The reaction mixture consisted of 5 µL template cDNA, 12.5 µL ROX SYBR Master Mix blue dTTP (Eurogentec, Seraing, Belgium), 1 µL of both forward and reverse species-specific primer (final primer concentration of 400 nM) and 5.5 µL distilled water. Assay conditions were an initial 2 min at 50 °C, followed by a 10-min denaturation step at 95 °C, 45 cycles of 15 s at 95 °C and 60 s at 60 °C. Sequences of each primer pair are described elsewhere^44^. Finally, data were determined as a function of the threshold cycle (CT) values and relative virulence gene expression was calculated according to the ΔΔCT method (2^−(ΔCTexp − ΔCTcontrol)^).

### Biofilm challenge of cells

Immortalized human oral keratinocytes (HOK-18A) were cultured as described previously^71^. The HOK-18A cell line is an immortalized human oral keratinocyte cell line derived from normal human oral keratinocyte cells originating from healthy donors^72^. The HOK-18A cell line used in this study was kindly gifted in the past by the late Prof. Susan Ann Kinder Haake (Section of Periodontics, School of Dentistry, UCLA, Los Angeles, California, USA). Sterile silicone rings (Peleman BVBA, Wilsele, Belgium) were placed at the bottom of the wells of 24-well plates, after which the HOK-18A cultures were seeded and grown until confluence. Biofilm coated disks were dip-rinsed in PBS (pH 7.4) to remove unattached cells and subsequently placed on top of the silicon rings with the biofilm facing the cell monolayer. The silicon rings ensured a fixed distance of 1 mm between the cell monolayer and the biofilm. After 2 h of contact (5% CO_2_, 37 °C, 170 rpm), the disks and rings were carefully removed and cells were washed twice with PBS (pH 7.4). Fresh cell culture medium containing 0.1 mg/mL gentamycin was added, followed by incubation (5% CO_2_, 37 °C, 170 rpm) for 2 h. After 2 h, cells were harvested and cellular RNA was extracted with the RNeasy kit (Qiagen, Hilden, Germany) according to the manufacturer’s protocol. Conversion of RNA to cDNA and determination of the relative expression of inflammatory mediator genes was determined as described above using the cellular housekeeping gene β-actin. In addition, supernatant of the cell cultures was collected and analysed through enzyme-linked immunosorbent assay (ELISA) to detect CXCL8 (interleukin-8, IL-8) using the Human IL-8 ELISA Kit (Thermo Fisher Scientific, Waltham, USA) according to the manufacturer’s instructions. All experiments were repeated on three different days.

### Statistical analysis

GraphPad Prism version 7.04 for Windows (GraphPad Software, La Jolla, California, USA) was used for statistical analysis. Normality of the residuals was assessed by means of a Shapiro–Wilk test and a normal quantile plot. For all experiments, first comparisons between the three substrate conditions (NADM, NADG, d-(+)-mannose) and the control were set up and analysed through a one-way ANOVA with a confidence level of 95% and a correction for simultaneous hypothesis testing was applied according to Dunnett. Differences between substrate conditions were only considered to be relevant if each of the conditions were also significantly different from the control condition. Such comparisons between substrate conditions were analysed either through a one-way ANOVA with a confidence level of 95% and a correction for simultaneous hypothesis testing according to Tukey (in the case of a comparison between 3 substrate conditions) or through an unpaired two-tailed t-test with a confidence level of 95% (in the case of a comparison between 2 substrate conditions).

## Supplementary Information

Supplementary Information.

## Data Availability

The authors declare that all data supporting the findings of this study are available within the paper and its supplementary information files.

## References

[1] Darveau RP (2010). Periodontitis: A polymicrobial disruption of host homeostasis. Nat. Rev. Microbiol..

[2] Hajishengallis G (2011). Low-abundance biofilm species orchestrates inflammatory periodontal disease through the commensal microbiota and complement. Cell Host Microbe.

[3] Marsh PD (1994). Microbial ecology of dental plaque and its significance in health and disease. Adv. Dent. Res..

[4] Marsh PD (2003). Are dental diseases examples of ecological catastrophes?. Microbiology.

[5] Mira A, Simon-Soro A, Curtis MA (2017). Role of microbial communities in the pathogenesis of periodontal diseases and caries. J. Clin. Periodontol..

[6] Roberts FA, Darveau RP (2015). Microbial protection and virulence in periodontal tissue as a function of polymicrobial communities: Symbiosis and dysbiosis. Periodontol 2000.

[7] Rosier BT, Marsh PD, Mira A (2018). Resilience of the oral microbiota in health: Mechanisms that prevent dysbiosis. J. Dent. Res..

[8] Silva N (2015). Host response mechanisms in periodontal diseases. J. Appl. Oral Sci..

[9] Hajishengallis G, Lamont RJ (2012). Beyond the red complex and into more complexity: The polymicrobial synergy and dysbiosis (PSD) model of periodontal disease etiology. Mol. Oral. Microbiol..

[10] Marsh PD (2010). Controlling the oral biofilm with antimicrobials. J. Dent..

[11] Chatzigiannidou I, Teughels W, Van de Wiele T, Boon N (2020). Oral biofilms exposure to chlorhexidine results in altered microbial composition and metabolic profile. NPJ Biofilms Microbiomes.

[12] Cieplik F (2019). Resistance toward chlorhexidine in oral bacteria—Is there cause for concern?. Front. Microbiol..

[13] Verspecht T (2019). Development of antiseptic adaptation and cross-adapatation in selected oral pathogens in vitro. Sci. Rep..

[14] Haque M, Sartelli M, Haque SZ (2019). Dental infection and resistance-global health consequences. Dent. J. (Basel).

[15] Hill C (2014). Expert consensus document. The International Scientific Association for Probiotics and Prebiotics consensus statement on the scope and appropriate use of the term probiotic. Nat. Rev. Gastroenterol. Hepatol..

[16] Gruner D, Paris S, Schwendicke F (2016). Probiotics for managing caries and periodontitis: Systematic review and meta-analysis. J. Dent..

[17] Teughels W (2013). Clinical and microbiological effects of Lactobacillus reuteri probiotics in the treatment of chronic periodontitis: A randomized placebo-controlled study. J. Clin. Periodontol..

[18] Gibson GR (2017). Expert consensus document: The International Scientific Association for Probiotics and Prebiotics (ISAPP) consensus statement on the definition and scope of prebiotics. Nat. Rev. Gastroenterol. Hepatol..

[19] Koopman JE (2017). Changes in the oral ecosystem induced by the use of 8% arginine toothpaste. Arch. Oral Biol..

[20] Koopman JE (2015). Stability and resilience of oral microcosms toward acidification and Candida outgrowth by arginine supplementation. Microb Ecol..

[21] Slomka V (2017). Nutritional stimulation of commensal oral bacteria suppresses pathogens: The prebiotic concept. J. Clin. Periodontol..

[22] Slomka V (2018). Oral prebiotics and the influence of environmental conditions in vitro. J. Periodontol..

[23] Wolff M (2013). In vivo effects of a new dentifrice containing 1.5% arginine and 1450 ppm fluoride on plaque metabolism. J. Clin. Dent..

[24] Verspecht T (2021). Potential prebiotic substrates modulate composition, metabolism, virulence and inflammatory potential of an in vitro multi-species oral biofilm. J. Oral Microbiol..

[25] Moye ZD, Burne RA, Zeng L (2014). Uptake and metabolism of N-acetylglucosamine and glucosamine by Streptococcus mutans. Appl. Environ. Microbiol..

[26] Revilla-Nuin B (2002). Transport of N-acetyl-d-mannosamine and N-acetyl-d-glucosamine in *Escherichia coli* K1: Effect on capsular polysialic acid production. FEBS Lett..

[27] Devine DA, Marsh PD (2009). Prospects for the development of probiotics and prebiotics for oral applications. J. Oral Microbiol..

[28] Davani-Davari D (2019). Foods.

[29] Palframan RJ, Gibson GR, Rastall RA (2002). Effect of pH and dose on the growth of gut bacteria on prebiotic carbohydrates in vitro. Anaerobe.

[30] Ruszkowski J, Witkowski JM (2019). Lactulose: Patient- and dose-dependent prebiotic properties in humans. Anaerobe.

[31] Aas JA, Paster BJ, Stokes LN, Olsen I, Dewhirst FE (2005). Defining the normal bacterial flora of the oral cavity. J. Clin. Microbiol..

[32] Dewhirst FE (2010). The human oral microbiome. J. Bacteriol..

[33] Zaura E, Keijser BJ, Huse SM, Crielaard W (2009). Defining the healthy "core microbiome" of oral microbial communities. BMC Microbiol..

[34] Davis LM, Martinez I, Walter J, Hutkins R (2010). A dose dependent impact of prebiotic galactooligosaccharides on the intestinal microbiota of healthy adults. Int. J. Food Microbiol..

[35] Takahashi N (2005). Microbial ecosystem in the oral cavity: Metabolic diversity in an ecological niche and its relationship with oral diseases. Int. Congr. Ser..

[36] Fernandez YMM, Exterkate RAM, Buijs MJ, Crielaard W, Zaura E (2017). Effect of mouthwashes on the composition and metabolic activity of oral biofilms grown in vitro. Clin. Oral Investig..

[37] Levy R, Borenstein E (2013). Metabolic modeling of species interaction in the human microbiome elucidates community-level assembly rules. Proc. Natl. Acad. Sci. USA.

[38] Takahashi N (2015). Oral microbiome metabolism: From "Who Are They?" to "What Are They Doing?". J. Dent. Res..

[39] Amir SM, Barker SA, Woodbury SA (1965). N-Acetylmannosamine digestion by human oral bacteria. Nature.

[40] Gualdi L (2012). Regulation of neuraminidase expression in Streptococcus pneumoniae. BMC Microbiol..

[41] How MJ, Long VJ, Woodbury SA (1967). Digestion of N-acetylmannosamine and n-acetylneuraminic acid by human oral bacteria. Nature.

[42] Qiqiang L, Huanxin M, Xuejun G (2012). Longitudinal study of volatile fatty acids in the gingival crevicular fluid of patients with periodontitis before and after nonsurgical therapy. J. Periodontal Res..

[43] Tsuda H, Ochiai K, Suzuki N, Otsuka K (2010). Butyrate, a bacterial metabolite, induces apoptosis and autophagic cell death in gingival epithelial cells. J. Periodontal Res..

[44] Herrero ER (2018). Dysbiotic biofilms deregulate the periodontal inflammatory response. J. Dent. Res..

[45] Dahlen G, Basic A, Bylund J (2019). Importance of virulence factors for the persistence of oral bacteria in the inflamed gingival crevice and in the pathogenesis of periodontal disease. J. Clin. Med..

[46] Duran-Pinedo AE, Yost S, Frias-Lopez J (2015). Small RNA transcriptome of the oral microbiome during periodontitis progression. Appl. Environ. Microbiol..

[47] Lamont RJ, Hajishengallis G (2015). Polymicrobial synergy and dysbiosis in inflammatory disease. Trends Mol. Med..

[48] Lamont RJ, Koo H, Hajishengallis G (2018). The oral microbiota: Dynamic communities and host interactions. Nat. Rev. Microbiol..

[49] Arimatsu K (2014). Oral pathobiont induces systemic inflammation and metabolic changes associated with alteration of gut microbiota. Sci. Rep..

[50] Chow J, Tang H, Mazmanian SK (2011). Pathobionts of the gastrointestinal microbiota and inflammatory disease. Curr. Opin. Immunol..

[51] Cugini C, Klepac-Ceraj V, Rackaityte E, Riggs JE, Davey ME (2013). Porphyromonas gingivalis: keeping the pathos out of the biont. J. Oral Microbiol..

[52] Darveau RP, Hajishengallis G, Curtis MA (2012). Porphyromonas gingivalis as a potential community activist for disease. J. Dent. Res..

[53] Han YW (2015). Fusobacterium nucleatum: A commensal-turned pathogen. Curr. Opin. Microbiol..

[54] Norskov-Lauritsen N, Claesson R, Birkeholm Jensen A, Aberg CH, Haubek D (2019). Aggregatibacter actinomycetemcomitans: Clinical significance of a pathobiont subjected to ample changes in classification and nomenclature. Pathogens.

[55] Duran-Pinedo AE (2014). Community-wide transcriptome of the oral microbiome in subjects with and without periodontitis. ISME J..

[56] Yost S, Duran-Pinedo AE, Teles R, Krishnan K, Frias-Lopez J (2015). Functional signatures of oral dysbiosis during periodontitis progression revealed by microbial metatranscriptome analysis. Genome Med..

[57] Kastbjerg VG, Larsen MH, Gram L, Ingmer H (2010). Influence of sublethal concentrations of common disinfectants on expression of virulence genes in Listeria monocytogenes. Appl. Environ. Microbiol..

[58] Knudsen GM, Holch A, Gram L (2012). Subinhibitory concentrations of antibiotics affect stress and virulence gene expression in Listeria monocytogenes and cause enhanced stress sensitivity but do not affect Caco-2 cell invasion. J. Appl. Microbiol..

[59] Liu Q, Zheng Z, Kim W, Burgwyn Fuchs B, Mylonakis E (2018). Influence of subinhibitory concentrations of NH125 on biofilm formation & virulence factors of *Staphylococcus aureus*. Future Med. Chem..

[60] Goutoudi P, Diza E, Arvanitidou M (2012). Effect of periodontal therapy on crevicular fluid interleukin-6 and interleukin-8 levels in chronic periodontitis. Int. J. Dent..

[61] Groeger S, Meyle J (2019). Oral mucosal epithelial cells. Front. Immunol..

[62] Ferrero MA, Reglero A, Fernandez-Lopez M, Ordas R, Rodriguez-Aparicio LB (1996). N-acetyl-d-neuraminic acid lyase generates the sialic acid for colominic acid biosynthesis in *Escherichia coli* K1. Biochem. J..

[63] Plumbridge J, Vimr E (1999). Convergent pathways for utilization of the amino sugars N-acetylglucosamine, N-acetylmannosamine, and N-acetylneuraminic acid by *Escherichia coli*. J. Bacteriol..

[64] Riga MS, Llado-Pelfort L, Artigas F, Celada P (2018). The serotonin hallucinogen 5-MeO-DMT alters cortico-thalamic activity in freely moving mice: Regionally-selective involvement of 5-HT1A and 5-HT2A receptors. Neuropharmacology.

[65] Tokunaga E, Yamamoto T, Ito E, Shibata N (2018). Understanding the thalidomide chirality in biological processes by the self-disproportionation of enantiomers. Sci. Rep..

[66] Zuloaga DG, Heck AL, De Guzman RM, Handa RJ (2020). Roles for androgens in mediating the sex differences of neuroendocrine and behavioral stress responses. Biol. Sex Differ..

[67] Alvarez G, Gonzalez M, Isabal S, Blanc V, Leon R (2013). Method to quantify live and dead cells in multi-species oral biofilm by real-time PCR with propidium monoazide. AMB Express.

[68] Exterkate RA, Crielaard W, Ten Cate JM (2010). Different response to amine fluoride by Streptococcus mutans and polymicrobial biofilms in a novel high-throughput active attachment model. Caries Res..

[69] Herrero ER (2016). Dysbiosis by neutralizing commensal mediated inhibition of pathobionts. Sci. Rep..

[70] Vandecasteele SJ, Peetermans WE, Merckx R, Van Ranst M, Van Eldere J (2002). Use of gDNA as internal standard for gene expression in staphylococci in vitro and in vivo. Biochem. Biophys. Res. Commun..

[71] Sliepen I (2009). Microbial interactions influence inflammatory host cell responses. J. Dent. Res..

[72] Huang GT, Haake SK, Kim JW, Park NH (1998). Differential expression of interleukin-8 and intercellular adhesion molecule-1 by human gingival epithelial cells in response to Actinobacillus actinomycetemcomitans or Porphyromonas gingivalis infection. Oral Microbiol. Immunol..

