# Supplementary material for: Comparison of the modulatory effects of three structurally similar potential prebiotic substrates on an in vitro multi-species oral biofilm
Source: Sci Rep. 2021 Jul 22;11:15033. doi: 10.1038/s41598-021-94510-z (PMC8298493; doi:10.1038/s41598-021-94510-z)
Supplement: Supplementary file 1 — Supplementary Information. [file 41598_2021_94510_MOESM1_ESM.pdf]

SUPPLEMENTARY INFORMATION

Comparison of the modulatory effects of three structurally similar potential prebiotic substrates on an in vitro multi-species oral biofilm

Tim Verspecht[1,2], Wannes Van Holm[1,2], Nico Boon[2], Kristel Bernaerts[3], Carlo A Daep[4], Naiera Zayed[1,2,5], Marc Quirynen[1,6], Wim Teughels[1,6 *]

[1] Department of Oral Health Sciences, University of Leuven (KU Leuven), Kapucijnenvoer 33, 3000 Leuven, Belgium.

[2] Center for Microbial Ecology and Technology (CMET), Ghent University (UGent), Coupure Links 653, 9000 Gent, Belgium.

[3] Bio- and Chemical Systems Technology, Reactor Engineering and Safety, Department of Chemical Engineering, University of Leuven (KU Leuven), Leuven Chem&Tech, Celestijnenlaan 200F (bus 2424), 3001 Leuven, Belgium.

[4] Colgate-Palmolive Technology Center, 909 River Road, Piscataway, NJ 08854, USA.

[5] Faculty of Pharmacy, Menoufia University, Egypt.

[6] Dentistry, University Hospitals Leuven, Kapucijnenvoer 33, 3000 Leuven, Belgium.

E-mail addresses authors: tim.verspecht@kuleuven.be, wannes.vanholm@kuleuven.be, nico.boon@ugent.be, kristel.bernaerts@kuleuven.be, carlo_daep@colpal.com, naiera.zayed@kuleuven.be, marc.quirynen@med.kuleuven.be, wim.teughels@med.kuleuven.be

[*] **Corresponding author:** Wim Teughels, KU Leuven, Department of Oral Health Sciences, Kapucijnenvoer 33, 3000 Leuven, Belgium. Phone: +32 (0)16 332 505, fax: +32 (0)16 332 484. E-mail: wim.teughels@med.kuleuven.be

Supplementary Table S1 Comparison of the effects of repeated rinsing with NADM, NADG or D-(+)-mannose at 1 M on multi-species biofilm composition (absolute abundances)

	log(Geq/mL)			
**1 M**	control	NADM	NADG	D-mannose
Aa	8.84 ± 0.24$^{\#2,3}$	7.84 ± 0.53	6.6 ± 0.93*	6.07 ± 0.47*
Fn	8.92 ± 0.49$^{\#2,3}$	8.14 ± 0.44	7.09 ± 0.30*	6.59 ± 0.36*
Pg	6.49 ± 0.80$^{\#2,3}$	5.42 ± 0.23	4.00 ± 0.39$^{*,\#3}$	2.75 ± 0.08$^{*,\#2}$
Pi	8.41 ± 0.42$^{\#2}$	9.07 ± 0.06	6.93 ± 0.62*	7.80 ± 0.57
S. mutans	8.57 ± 0.40$^{\#1}$	7.41 ± 0.44*	8.95 ± 0.38	8.60 ± 0.09
S. sobrinus	5.91 ± 0.32$^{\#2}$	6.05 ± 0.23	4.16 ± 0.44*	5.44 ± 0.31
**1 M**	control	NADM	NADG	D-mannose
An	6.74 ± 0.33$^{\#3}$	6.73 ± 0.39	6.52 ± 0.20	5.54 ± 0.24*
Av	8.12 ± 0.70$^{\#3}$	8.35 ± 0.30	6.92 ± 0.45	6.40 ± 0.48*
S. gord.	8.52 ± 0.27	8.94 ± 0.32	9.38 ± 0.40	9.19 ± 0.39
S. mitis	<LOD	<LOD	<LOD	<LOD
S. oralis	6.96 ± 0.27$^{\#1,2}$	9.46 ± 0.24$^{*,\#2}$	7.97 ± 0.31$^{*,\#1}$	6.83 ± 0.09
S. sal.	3.23 ± 0.50	3.34 ± 0.55	4.38 ± 1.23	3.63 ± 0.90
S. sang.	7.70 ± 0.61	9.10 ± 0.29	8.11 ± 0.68	7.39 ± 0.89
Vp	10.17 ± 0.33	10.76 ± 0.09	10.57 ± 0.24	10.18 ± 0.09

Absolute abundances of bacterial species are shown as mean ± SD (n = 3) logarithmic values of the genome equivalents per millilitre (log(Geq/mL)). All substrates were dissolved in PBS at a concentration of 1 M. Statistically significantly different values are marked with ‘*’ (vs. control (PBS)), ‘$^{\#1}$’ (vs. NADM), ‘$^{\#2}$’ (vs. NADG), ‘$^{\#3}$’ (vs. D-(+)-mannose) ($P < 0.05$). Statistically significantly different values between treatment conditions were only considered to be relevant if each condition was also significantly different from the control condition and only such differences are indicated.

Aa: *A. actinomycetemcomitans*; *Fn*: *F. nucleatum*; *Pg*: *P. gingivalis*; *Pi*: *P. intermedia*; *An*: *A. naeslundii*; *Av*: *A. viscosus*; *S. gord.*: *S. gordonii*; *S. sal.*: *S. salivarius*; *S. sang.*: *S. sanguinis*; *Vp*: *V. parvula*; LOD: limit of detection (=2.65 log(Geq/mL)); NADM: N-acetyl-D-mannosamine; NADG: N-acetyl-D-glucosamine.

Supplementary Table S2 Comparison of the effects of repeated rinsing with NADM, NADG or D-(+)-mannose at ~0.05 M (1%$_{(w/v)}$) on multi-species biofilm composition (absolute abundances)

	log(Geq/mL)			
**1%**	control	NADM	NADG	D-mannose
Aa	9.49 ± 0.25	9.51 ± 0.05	9.11 ± 0.20	9.02 ± 0.11
Fn	9.71 ± 0.11$^{\#3}$	9.60 ± 0.22	9.41 ± 0.19	8.66 ± 0.29*
Pg	7.27 ± 0.20	7.06 ± 0.30	7.14 ± 0.28	7.08 ± 0.17
Pi	8.02 ± 0.24	7.48 ± 0.40	8.17 ± 0.22	8.06 ± 0.19
S. mutans	7.48 ± 0.13$^{\#1}$	7.01 ± 0.05*	7.83 ± 0.23	7.34 ± 0.12
S. sobrinus	5.16 ± 0.07	5.24 ± 0.27	4.87 ± 0.22	5.28 ± 0.44
**1%**	control	NADM	NADG	D-mannose
An	7.58 ± 0.13	7.60 ± 0.22	7.71 ± 0.12	7.59 ± 0.05
Av	8.01 ± 0.44	7.73 ± 0.09	7.83 ± 0.31	8.00 ± 0.17
S. gord.	8.33 ± 0.18	8.40 ± 0.10	8.33 ± 0.06	8.29 ± 0.11
S. mitis	<LOD	<LOD	<LOD	3.00 ± 0.49
S. oralis	6.90 ± 0.20	7.49 ± 0.30	7.11 ± 0.14	6.27 ± 0.21
S. sal.	2.86 ± 0.30	2.90 ± 0.36	2.65 ± 0.00	2.89 ± 0.35
S. sang.	8.37 ± 0.25	8.62 ± 0.39	8.4 ± 0.11	8.19 ± 0.11
Vp	10.07 ± 0.16	10.05 ± 0.17	9.77 ± 0.19	9.93 ± 0.20

Absolute abundances of bacterial species are shown as mean ± SD (n = 3) logarithmic values of the genome equivalents per millilitre (log(Geq/mL)). All substrates were dissolved in PBS at a concentration of ~0.05 M (1%$_{(w/v)}$) (corresponding molar concentrations: 45 mM (NADM and NADG) and 56 mM (D-(+)-mannose). Statistically significantly different values are marked with '*' (vs. control (PBS)), '$^{\#1}$' (vs. NADM), '$^{\#2}$' (vs. NADG), '$^{\#3}$' (vs. D-(+)-mannose) ($P < 0.05$). Statistically significantly different values between treatment conditions were only considered to be relevant if each condition was also significantly different from the control condition and only such differences are indicated.

Aa: *A. actinomycetemcomitans*; *Fn*: *F. nucleatum*; *Pg*: *P. gingivalis*; *Pi*: *P. intermedia*; *An*: *A. naeslundii*; *Av*: *A. viscosus*; *S. gord.*: *S. gordonii*; *S. sal.*: *S. salivarius*; *S. sang.*: *S. sanguinis*; *Vp*: *V. parvula*; LOD: limit of detection (=2.65 log(Geq/mL)); NADM: N-acetyl-D-mannosamine; NADG: N-acetyl-D-glucosamine.

Supplementary Table S3 Comparison of the effects of repeated rinsing with NADM, NADG or D-(+)-mannose on multi-species biofilm organic acid production/consumption

	organic acid production/consumption (mg/L)			
**1 M**	control	NADM	NADG	D-mannose
lactate	125 ± 0 (-)	125 ± 0 (-)	125 ± 0 (-)	125 ± 0 (-)
formate	384 ± 19[#1]	792 ± 138[*]	352 ± 18	398 ± 12
acetate	3779 ± 305[#1,3]	3084 ± 174[*,#3]	3815 ± 77	1934 ± 56[*,#1]
propionate	2094 ± 132[#2,3]	2052 ± 54	2750 ± 40[*]	2821 ± 127[*]
butyrate	1870 ± 93[#1,2,3]	541 ± 52[*,#2,3]	1255 ± 51[*,#1,3]	86 ± 15[*,#1,2]
**1%$_{(w/v)}$**	control	NADM	NADG	D-mannose
lactate	122 ± 5 (-)	125 ± 0 (-)	125 ± 0 (-)	125 ± 0 (-)
acetate	345 ± 63	396 ± 11	379 ± 10	352 ± 24
formate	3954 ± 539	3768 ± 174	4315 ± 198	3717 ± 114
propionate	2077 ± 234	1891 ± 79	2205 ± 89	1962 ± 28
butyrate	2129 ± 240	1989 ± 44	2238 ± 59	1985 ± 37

Organic acid levels detected in the supernatants of substrate-treated multi-species biofilms are shown as mean ± SD (n = 3) values (mg/L). Values accompanied by a negative sign '(-)' indicate a net decrease (consumption), all other values indicate a net increase (production) of organic acid. Substrates were dissolved in PBS at a concentration of 1 M (**upper part**) or ~0.05 M (1%$_{(w/v)}$) (**lower part**) (corresponding molar concentrations: 45 mM (NADM and NADG) and 56 mM (D-(+)-mannose).

Statistically significantly different values are marked with '*' (vs. control (PBS)), '[#1]' (vs. NADM), '[#2]' (vs. NADG), '[#3]' (vs. D-(+)-mannose) ($P < 0.05$). Statistically significantly different values between treatment conditions were only considered to be relevant if each condition was also significantly different from the control condition and only such differences are indicated. NADM: N-acetyl-D-mannosamine; NADG: N-acetyl-D-glucosamine.

Supplementary Table S4 Overview of virulence genes and associated functions of the corresponding virulence factors

A. actinomycetemcomitans	
**Virulence gene**	**Associated function of corresponding virulence factor**
Fimbriae subunit *flp* gene	Attachment to host tissues through fimbriae [1]
Autotransporter adhesin *aae* gene	Attachment to epithelial and endothelial cells [2]
Tetraphosphatase *apaH* gene	Invasion and colonization of non-phagocytic cells [3]
Cytolethal distending toxin *cdtB* gene	Immune modulation by induction of apoptosis of T lymphocytes and non-proliferative monocytic cells [3]
Extracellular matrix adhesin protein A *emaA* gene	Biofilm formation by mediating collagen binding [1]
Leukotoxin *ltxA* gene	Immune evasion through lysis of human lymphocytes and monocytes [4]
Outer membrane protein *omp100* gene	Adherence to epithelial cells and highly immunogenic auto-transported protein [5]
Outer membrane protein *omp29* gene	Invasion of non-phagocytic cells and highly immunogenic auto-transported protein [3]
orf859 gene	Intracellular survival [3]
Extracellular polysaccharide synthesis *pgA* gene	Synthesis of a linear polysaccharide with an important role in aggregation and biofilm formation [6]
Virulence associate protein *vapA* gene	Direct role in virulence and necessary for acquisition of other virulence factors [3]
Virulence plasmid protein *vppA* gene	Direct role in virulence and necessary for acquisition of other virulence factors [3]
P. gingivalis	
**Virulence gene**	**Associated function of corresponding virulence factor**
Lysine-specific cysteine proteinase *kgp* gene	Disturbance of host defense systems, activation of the blood clotting system and several other processes, potent fibrin/fibrinogen degrading enzyme [7]
FimA type II fimbrilin *fimA* gene	Attachment to oral surfaces [8]
Collagenase *partC* gene	Host tissue colonization and degradation, collagenase precursor [9]
Arginine-specific cysteine proteinase *rgpB* gene	Disturbance of host defense systems, activation of the blood clotting system and several other processes, potent fibrin/fibrinogen degrading enzyme [7]
F. nucleatum	
**Virulence gene**	**Associated function of corresponding virulence factor**
Butyrate-acetoacetate CoA-transferase subunit B gene	Butyrate metabolism and thus involvement in inflammatory processes and cell killing that occur during periodontal disease [10,11]

Outer membrane protein A *ompA* gene	Cell wall and membrane envelope biogenesis, highly immunogenic [12]
EF-G elongation factor G *EF-G* gene	mRNA translation, highly immunogenic [13]
ABC transporter permease gene	Membrane transport, highly immunogenic [13]
Transposase gene	Role in virulence and necessary for the acquisition of other virulence factors [14]
Hemolysin gene	Lysis of erythrocytes, production of iron and creation of anaerobic environment [13]
Hemin receptor gene	Hemin uptake, highly immunogenic [13]
P. intermedia	
**Virulence gene**	**Associated function of corresponding virulence factor**
Invasin *adpC* gene	Cell adhesion and invasion [15]
Chaperone *clpB* gene	Resistance to stressful host environments, heat shock protein [16]
Chaperone *dnaK* gene	Resistance to stressful host environments, heat shock protein [16]
Chaperone *dnaJ* gene	Resistance to stressful host environments, heat shock protein [16]
Extra-cytoplasmatic function-subfamily sigma factor *ecf* gene	Exopolysaccharide production during stress response and biofilm formation [17]
Chaperone *groES* gene	Resistance to stressful host environments, heat shock protein [16]
Heat shock protein *htpG* gene	Resistance to stressful host environments, heat shock protein [16]
Polysialic acid transport protein *kpsD* gene	Polysaccharide production during stress response and biofilm formation [17]
Interpain A *inpA* gene	Immune evasion by degradation of complement factors [18]
Hemagglutinin *phg* gene	Hemagglutination [19]

References Supplementary Table S4

1. Danforth, D. R., Tang-Siegel, G., Ruiz, T. & Mintz, K. P. A Nonfimbrial Adhesin of Aggregatibacter actinomycetemcomitans Mediates Biofilm Biogenesis. Infect Immun 87, doi:10.1128/IAI.00704-18 (2019).
2. Fine, D. H. et al. Mapping the epithelial-cell-binding domain of the Aggregatibacter actinomycetemcomitans autotransporter adhesin Aae. Microbiology 156, 3412-3420, doi:10.1099/mic.0.037606-0 (2010).
3. Umeda, J. E., Longo, P. L., Simionato, M. R. & Mayer, M. P. Differential transcription of virulence genes in Aggregatibacter actinomycetemcomitans serotypes. J Oral Microbiol 5, doi:10.3402/jom.v5i0.21473 (2013).
4. Dileepan, T., Kachlany, S. C., Balashova, N. V., Patel, J. & Maheswaran, S. K. Human CD18 is the functional receptor for Aggregatibacter actinomycetemcomitans leukotoxin. Infect Immun 75, 4851-4856, doi:10.1128/IAI.00314-07 (2007).

5. Asakawa, R. et al. Outer membrane protein 100, a versatile virulence factor of Actinobacillus actinomycetemcomitans. Mol Microbiol 50, 1125-1139, doi:10.1046/j.1365-2958.2003.03748.x (2003).
6. Hisano, K. et al. The pga gene cluster in Aggregatibacter actinomycetemcomitans is necessary for the development of natural competence in Ca(2+) -promoted biofilms. Mol Oral Microbiol 29, 79-89, doi:10.1111/omi.12046 (2014).
7. Imamura, T. The role of gingipains in the pathogenesis of periodontal disease. J Periodontol 74, 111-118, doi:10.1902/jop.2003.74.1.111 (2003).
8. Xie, H., Kozlova, N. & Lamont, R. J. Porphyromonas gingivalis genes involved in fimA regulation. Infect Immun 72, 651-658, doi:10.1128/iai.72.2.651-658.2004 (2004).
9. Houle, M. A., Grenier, D., Plamondon, P. & Nakayama, K. The collagenase activity of Porphyromonas gingivalis is due to Arg-gingipain. FEMS Microbiol Lett 221, 181-185, doi:10.1016/S0378-1097(03)00178-2 (2003).
10. Tsuda, H., Ochiai, K., Suzuki, N. & Otsuka, K. Butyrate, a bacterial metabolite, induces apoptosis and autophagic cell death in gingival epithelial cells. J Periodontal Res 45, 626-634, doi:10.1111/j.1600-0765.2010.01277.x (2010).
11. Vital, M., Howe, A. C. & Tiedje, J. M. Revealing the bacterial butyrate synthesis pathways by analyzing (meta)genomic data. mBio 5, e00889, doi:10.1128/mBio.00889-14 (2014).
12. Bakken, V., Aaro, S., Hofstad, T. & Vasstrand, E. N. Outer membrane proteins as major antigens of Fusobacterium nucleatum. FEMS Microbiol Immunol 1, 473-483, doi:10.1111/j.1574-6968.1989.tb02438.x (1989).
13. Lee, H. R. et al. In-vivo-induced antigenic determinants of Fusobacterium nucleatum subsp. nucleatum. Mol Oral Microbiol 26, 164-172, doi:10.1111/j.2041-1014.2010.00594.x (2011).
14. McKay, T. L., Ko, J., Bilalis, Y. & DiRienzo, J. M. Mobile genetic elements of Fusobacterium nucleatum. Plasmid 33, 15-25, doi:10.1006/plas.1995.1003 (1995).
15. Iyer, D. et al. AdpC is a Prevotella intermedia 17 leucine-rich repeat internalin-like protein. Infect Immun 78, 2385-2396, doi:10.1128/IAI.00510-09 (2010).
16. Neckers, L. & Tatu, U. Molecular chaperones in pathogen virulence: emerging new targets for therapy. Cell Host Microbe 4, 519-527, doi:10.1016/j.chom.2008.10.011 (2008).
17. Yamanaka, T. et al. Gene expression profile and pathogenicity of biofilm-forming Prevotella intermedia strain 17. Bmc Microbiol 9, 11, doi:10.1186/1471-2180-9-11 (2009).
18. Potempa, M. et al. Interpain A, a cysteine proteinase from Prevotella intermedia, inhibits complement by degrading complement factor C3. PLoS Pathog 5, e1000316, doi:10.1371/journal.ppat.1000316 (2009).
19. Okamoto, M., Maeda, N., Kondo, K. & Leung, K. P. Hemolytic and hemagglutinating activities of Prevotella intermedia and Prevotella nigrescens. FEMS Microbiol Lett 178, 299-304, doi:10.1111/j.1574-6968.1999.tb08691.x (1999).

Supplementary Table S5 Comparison of effects of repeated multi-species biofilm rinsing with NADM, NADG or D-(+)-mannose on multi-species biofilm inflammatory potential towards human oral keratinocytes

		relative fold change in inflammatory mediator gene expression		
**1 M**	**Genes**	**NADM**	**NADG**	**D-mannose**
	IL-1β	0.59 (0.30-1.14)	0.75 (0.55-1.02)	0.81 (0.59-1.12)
	IL-6	0.86 (0.34-2.18)	0.57 (0.46-0.71)	0.63 (0.41-0.97)
	IL-8	**0.10 (0.05-0.17)***	**0.14 (0.06-0.36)***	**0.12 (0.06-0.27)***
	MMP-8	**1.66 (0.82-3.35)***	1.28 (0.79-2.08)	1.25 (1.10-1.42)
	TNF-α	0.73 (0.64-0.83)	0.89 (0.59-1.34)	0.77 (0.37-1.60)
**1%$_{(w/v)}$**	**Genes**	**NADM**	**NADG**	**D-mannose**
	IL-1β	0.83 (0.66-1.04)	0.71 (0.38-1.33)	0.79 (0.43-1.48)
	IL-6	0.73 (0.48-1.13)	0.73 (0.58-0.93)	0.79 (0.60-1.06)
	IL-8	1.32 (1.14-1.53)	1.00 (0.78-1.27)	1.22 (0.79-1.86)
	MMP-8	0.62 (0.21-1.84)	0.52 (0.17-1.60)	**0.40 (0.27-0.58)***
	TNF-α	0.69 (0.48-1.00)	0.71 (0.28-1.35)	0.77 (0.48-1.23)

relative fold change values

<0.1	0.1-0.3	0.3-0.5	0.5-0.7	0.7-0.9	0.9-1.1	1.1-1.4	1.4-2.0	2.0-3.3	3.3-10	>10.0

Fold changes in inflammatory mediator gene expression from human oral keratinocytes (HOK-18A) exposed to substrate-treated multi-species biofilms were determined relative to the control through the $2^{-\Delta\Delta Ct}$ method and are shown as the geometric mean (C.I.) (n = 3) of the $2^{-\Delta\Delta Ct}$ values. All substrates were dissolved in PBS at a concentration of 1 M (**upper part**) or ~0.05 M (1%$_{(w/v)}$) (**lower part**) (corresponding molar concentrations: 45 mM (NADM and NADG) and 56 mM (D-(+)-mannose). Values between 0 and 1 represent relative downregulation, values >1 represent relative upregulation. Statistically significantly different fold changes relative to the control (PBS) that are <0.5 (>2-fold downregulated) or >1.5 (>1.5-fold upregulated) are considered biologically relevant and are shown in bold and marked with '*****' ($P < 0.05$). For such values, statistically significant differences between two treatment conditions are marked with '**#1**' (vs. NADM), '**#2**' (vs. NADG), '**#3**' (vs. D-(+)-mannose) and shown in bold ($P < 0.05$). Statistically significantly different values between treatment conditions were only considered relevant if each condition was also significantly different from the control condition and only such differences are indicated. Color code: magnitude of the fold change in virulence gene expression relative to the control. NADG: N-acetyl-D-glucosamine; C.I.: 95% confidence interval.

Supplementary Table S6 Comparison of the effects of repeated rinsing with NADM, NADG or D-(+)-mannose on multi-species biofilm inflammatory potential

	IL-8 levels (pg/mL)			
**1 M**	control	NADM	NADG	D-mannose
	21.43 ± 4.67[#1,2,3]	0.59 ± 0.61*	0.22 ± 0.32*	0.06 ± 0.04*
**1%$_{(w/v)}$**	control	NADM	NADG	D-mannose
	72.52 ± 19.2[#1]	30.32 ± 6.26*	54.75 ± 11.66	51.09 ± 13.89

IL-8 levels detected in the supernatants of human oral keratinocytes (HOK-18A) cultures exposed to substrate-treated multi-species biofilms are shown as mean ± SD (n = 3) values (pg/mL). Substrates were dissolved in PBS at a concentration of 1 M (**upper row**) or ~0.05 M (1%$_{(w/v)}$) (**lower row**) (corresponding molar concentrations: 45 mM (NADM and NADG) and 56 mM (D-(+)-mannose).

Statistically significantly different values are marked with '*****' (vs. control (PBS)), '[#1]' (vs. NADM), '[#2]' (vs. NADG), '[#3]' (vs. D-(+)-mannose) (P < 0.05). Statistically significantly different values between treatment conditions were only considered to be relevant if each condition was also significantly different from the control condition and only such differences are indicated. IL-8: interleukin-8; NADM: N-acetyl-D-mannosamine; NADG: N-acetyl-D-glucosamine.
